# Proteasomal Protein Degradation: Adaptation of Cellular Proteolysis With Impact on Virus—and Cytokine-Mediated Damage of Heart Tissue During Myocarditis

**DOI:** 10.3389/fimmu.2018.02620

**Published:** 2018-11-28

**Authors:** Antje Beling, Meike Kespohl

**Affiliations:** ^1^Charité—Universitätsmedizin Berlin, Corporate Member of Freie Universität Berlin, Humboldt-Universität zu Berlin, and Berlin Institute of Health (BIH), Institute of Biochemistry, Berlin, Germany; ^2^Deutsches Zentrum für Herz-Kreislauf-Forschung (DZHK), Partner Site Berlin, Berlin, Germany

**Keywords:** virus, myocarditis, proteasome, cytokine, immunopathology, heart failure

## Abstract

Viral myocarditis is an inflammation of the heart muscle triggered by direct virus-induced cytolysis and immune response mechanisms with most severe consequences during early childhood. Acute and long-term manifestation of damaged heart tissue and disturbances of cardiac performance involve virus-triggered adverse activation of the immune response and both immunopathology, as well as, autoimmunity account for such immune-destructive processes. It is a matter of ongoing debate to what extent subclinical virus infection contributes to the debilitating sequela of the acute disease. In this review, we conceptualize the many functions of the proteasome in viral myocarditis and discuss the adaptation of this multi-catalytic protease complex together with its implications on the course of disease. Inhibition of proteasome function is already highly relevant as a strategy in treating various malignancies. However, cardiotoxicity and immune-related adverse effects have proven significant hurdles, representative of the target's wide-ranging functions. Thus, we further discuss the molecular details of proteasome-mediated activity of the immune response for virus-mediated inflammatory heart disease. We summarize how the spatiotemporal flexibility of the proteasome might be tackled for therapeutic purposes aiming to mitigate virus-mediated adverse activation of the immune response in the heart.

## Introduction

Myocarditis and its debilitating sequela, inflammatory cardiomyopathy, are leading causes of heart failure and sudden cardiac death particularly in infants, children, and young adults (1) with viral infections being the most common trigger of non-ischemic myocardial inflammation in the Western world (2). Acute injury of the heart muscle upon viral infection stimulates infiltration of immune cells, aiming to support pathogen clearance and alleviate organ damage. However, this pathogen-induced immune response can result subsequently in overwhelming immunopathology or the development of auto-aggressive immunity against cardiac self-antigens. These processes comprise fibrotic scarring and cardiac remodeling (3, 4). Both the high mortality of acute viral myocarditis in childhood and the putative progression of acute myocarditis to chronic disease support the need to define precisely the underlying mechanisms.

Most of our knowledge on the pathology of viral myocarditis comes from infection with enteroviruses, in particular coxsackievirus B3 (CVB3) in mice. CVB belong to the picornavirus family and have a non-enveloped, icosahedral capsid surrounding a positive-strand RNA genome. CVB3 used to be among the most prevalent pathogens known to cause viral myocarditis in North America and Europe (3, 5). Infection of laboratory mouse strains mirrors the variable manifestation of the disease in man (6, 7) by causing susceptibility for cardiac pathogenesis to a highly varying degree. A certain genetic background determines both control of viral pathogens and the activation of deleterious immune response pathways (6, 8). Recent observational studies suggest that it is not primarily the presence and/or replicative activity of invading viruses in the myocardium that determines outcome. In fact, the virus-triggered abundance of infiltrating leukocytes is an independent risk factor for adverse outcome (9). Although it is indisputable that primary encounter of virus in the heart triggers death of cardiomyocytes, the pathogenic role of persisting viral genomes was poorly defined in the past. Recently, experimental mouse data demonstrated that persisting enteroviral RNAs do not actively contribute to ongoing myocardial disease after viral myocarditis (10).

Mice with high susceptibility to severe virus-induced inflammation are pre-disposed also to a loss of self-tolerance against cardiac proteins (11). Additionally, viral infection of cardiomyocytes can trigger auto-destructive activity of infiltrating cells, as well as, the formation of autoantibodies directed against antigens of cardiac origin (12, 13) further exaggerating heart tissue damage. Establishment of autoimmune myocarditis in mice by priming with cardiac antigens revealed that the same strains of inbred mice, who develop post-viral inflammatory heart tissue injury are also prone to autoimmune-triggered heart pathology. This indicates that the background genetics and involved immune response pathways for both diseases might be overlapping (13, 14). Others have reviewed in detail how type B coxsackievirus interacts with the innate and adaptive immune system and inflammatory responses (7, 15). Our primary interest herein is to discuss how cellular proteolysis by the proteasome affects the innate and adaptive immune response during CVB3-induced inflammatory damage of heart tissue, and our focus will broaden to the adaptation of this multi-catalytic protease in different cells during infection and inflammation. We will specifically discuss recent findings regarding the functional importance of a specific proteasome subtype expressed in hematopoietic cells and its possible implications for cytokine-mediated pathogenesis and therapeutic interference during viral myocarditis.

## The proteasome: a druggable multi-catalytic protease

Several avenues of research have implicated the ubiquitin-proteasome system (UPS) as a major regulator of cell signaling and transcription. It controls also antigen processing, apoptosis and cellular proliferation. The ubiquitination machinery tags degradation-prone proteins in a highly regulated system for processing by the proteasome. As an integral part of cellular proteostasis, proteasome-mediated protein degradation is the primary route for intracellular removal of misfolded, damaged, or short-lived proteins (16). Proteasomes are multi-subunit enzymes with a barrel-shaped structure and internal active sites are accessible through a gated pore (17, 18). Proteasome-destined cargoes are recognized by regulatory particles (19S regulator) associated with the proteasome core complex (20S proteasome). The recognition, de-ubiquitination, and unfolding of substrates in direct proximity to the gated entry channel made up of the outer α ring of the 20S proteasome is required for degradation (19). Peptide hydrolysis is restricted to three β subunits, β1, β2, and β5, within the interior of the 2-fold symmetric core 20S proteasome. In addition to the aforementioned functions of the proteasome, the UPS is also of particular importance under conditions of cellular stress, where a rapid elimination of unfolded and potentially toxic proteins is required to prevent formation of cytotoxic aggregates (16, 20). Restrained function of the UPS might lead to accumulation of harmful proteins to toxic levels, causing disease (21). Cells have several ways to meet such increased demand for protein turnover. In response to Interferon (IFN)-γ (22, 23), tumor necrosis factor (TNF)-α (24), doxorubicin (25), or H_2_O_2_ exposure (26), others and we demonstrated an increased abundance of the immunoproteasome (i-proteasome), a specific proteasome isoform that contains alternative catalytic subunits (β1i/low molecular weight protein (LMP) 2; β2i/multicatalytic endopeptidase complex (Mecl)-1, β5i/LMP7) (27). I-proteasomes at least partially replace their constitutively expressed standard proteasome counterpart in different tissues upon infection (28, 29). During viral myocarditis, the i-proteasome is upregulated strongly in heart tissue and its induction involves IFN-γ (30, 31), as well as, type 1 interferon (T1IFN)-mediated signaling (8). In the heart, i-proteasome formation results in increased peptide hydrolysis capacity (8). This adaptation within the proteolytic core of the 20S proteasome complex is advantageous since it contributes to maintenance of protein homeostasis during inflammation (23, 32, 33). I-proteasome assembly is very similar to the formation of the standard proteasome [reviewed recently by (34)]. Additional proteasome subtypes like the thymoproteasome with tissue-specific β5t subunit expression (35) and mixed proteasomes that contain only one (β5i) or two (β1i and β5i) of the three inducible catalytic subunits of the i-proteasome (36) contribute to the variety of proteasome-mediated proteolysis.

Both facts—the close vicinity of genes encoding β1i/LMP2, β5i/LMP7, and the transporter associated with antigen presentation (TAP) within the major histocompatibility (MHC) II region, as well as, the regulatory function of IFNγ for these molecules—were indicative for a specialized function of the i-proteasome during MHC class I antigen presentation (37). The finding that β1i/LMP2 and β5i/LMP7 enhance substrate cleavage after basic and hydrophobic amino acid residues further strengthened the notion for a specific role of the i-proteasome in the generation of antigenic peptides (22, 38). In fact, there are numerous examples for viral, bacterial, and parasitic pathogens for which *in vitro* peptide processing studies revealed facilitated MHC class I epitope liberation by the i-proteasome complex in comparison to lower epitope abundance upon processing of model polypeptides with the standard proteasome (39). This altered prevalence of antigenic peptide generation by the i-proteasome is attributed to different peptide cleavage site usage (40), and can elicit to altered CD8^+^ T cell-mediated immune surveillance also (41–46). Nevertheless, these findings appear to be restricted to a defined pool of immunodominant epitopes with no effect of the i-proteasome on other epitopes (28, 47, 48).

During the last three decades, the experimental landscape investigating i-proteasome biology substantially broadened with the availability of knockout mice lacking either single immunosubunits (47, 49) or a combinatory deletion of the three genes encoding β5i/LMP7, β1i/LMP2, and β2i/MECL-1 (45). Because deletion of a single i-proteasome subunit might be outweighed by increased formation of standard proteasome complexes (50), research on the i-proteasome improved further with the availability of i-proteasome subunit-selective inhibitors. Kisselev and Groettrup provided a detailed overview on inhibitors of the respective subunits of the immunoproteasome (51). Structure-guided optimization of such inhibitory compounds with subunit selectivity is actually an ongoing objective. Initially, development of i-proteasome-selective inhibitors was pursued with regard to the profound benefit in patients with multiple myeloma (MM) upon the implementation of non-selective proteasome inhibitors like bortezomib or carfilzomib (52–55). Despite their high efficacy for MM cells, targeting the proteasome in other organs like the heart constitutes a risk for heart failure (56). In comparison to heart tissue (57), MM cells are unique regarding the preferential expression of the i-proteasome in these cancer cells. Therefore, compounds with selective i-proteasome subunit specificity represent an alternative strategy for more selective tumor-directed targeting (54, 58). ONX 0914 initially known as PR957 is a potent i-proteasome-selective inhibitor that predominantly targets the β5i/LMP7 and to a lower degree the β1i/LMP2 i-proteasome subunit as well (29, 59). Beyond the tumor-suppressive potential of ONX 0914 (60, 61), pre-clinical research utilizing this compound and other i-proteasome-selective inhibitors revealed additional putative clinical scenarios, where such drugs might improve current medical treatment. Pioneering work by the Groettrup group and others highlighted the therapeutic potential of i-proteasome inhibitors for mitigation of autoimmune-driven inflammatory tissue damage (50, 59, 62–64). KZR-616—an ortholog of ONX 0914 with high selectivity for the human i-proteasome—passed successfully phase I trials and is now in phase II trials for patients with systemic lupus erythematosus. Since i-proteasome activity controls alloantibody production by B cells and influences processes resulting in T cell exhaustion, i-proteasome-selective compounds could be used to prevent allograft rejection upon organ transplantation as well (65, 66). All these recent reports shed light onto several previously unappreciated biological functions of the i-proteasome and support the requirement for a detailed overview on the pathological function of the proteasome during virus-induced inflammatory heart tissue injury.

## Viral entry, replication, and release: control mechanisms by the proteasome

Viruses subvert cellular processes to favor viral propagation. Given its central role in a wide range of cellular functions by maintaining a critical level of essential regulatory proteins, it is expected that the proteasome is involved in viral replication, and numerous examples have indeed been reported. Several viral proteins direct host-cell proteins to proteolytic degradation by the proteasome (67). Viruses have evolved e.g., by encoding specific ubiquitin ligase activity to employ the proteasome for degradation of host proteins that would impede viral growth. Since this review mainly focuses on the immunomodulatory function of the proteasome complex itself during manifestation of virus-mediated inflammatory damage of heart tissue, the reader is encouraged to refer to an excellent review recently provided by Honglin Luo on interactions between ubiquitin/ubiquitin family proteins and viral growth (68). Here, we will summarize examples of viruses with known cardiac tropism, where the proteasome complex is exploited for virus progeny formation and/or where inhibitors of proteasome activity affect viral replication (Table 1).

**Table 1:** Effect of the proteasome on the propagation of viral particles.

**Virus**	**Cell type**	**Treatment/condition**	**Effect on viral replication**	**Targeted step in life cycle of virus**	**References**
Adenovirus	HeLa cells	MG132	Reduced	Late gene expression	(69)
Mouse adenovirus^1^	C57BL/6 mice	LMP7^−/−^	No effect	n.r.	(31)
Coxsackie-virus B3 (CVB3)	Murine myxoma cell line HL-1	MG132, lactacystin	Reduced	Post entry	(70)
	A/J mice	MLN353	No effect	n.r.	(71)
	C57BL/6 mice	LMP7^−/−^	No effect	n.r.	(23)
	Murine embryonic cardiomyocytes	ONX 0914	No effect	n.r.	(72)
	C57BL/6 mice	ONX 0914	Increased cardiac titers		
	A/J mice	ONX 0914	No effect on cardiac titers		
	HeLa cells	PA28α/β siRNA	Increased	n.r.	(73)
	HeLa cells	PA28α/β overexpression	Reduced		
	Murine embryonic cardiomyocytes	PA28α/β^−/−^	Increased		
	C57BL/6 mice	PA28α/β^−/−^	No effect on cardiac titers		
Herpes simplex virus 1 (HSV-1)	Monkey kidney epithelial cells (Vero cells) Hamster ovary cells (CHO-cells)	MG132 epoxomicin lactacystin	Reduced	Virus entry/post penetration step	(74)
	HeLa derivative HEp-2	MG132, MG115, epoxomicin	Reduced	Immediate-early and late viral proteins	(75)
Human cytomegalo-virus (HCMV)	Human embryonic lung fibroblasts	MG132	Reduced	All stages of viral replication	(76)
	Human embryonic lung fibroblasts	MG132	Reduced	Immediate early protein synthesis	(77)
Human immuno-deficiency virus 1/2 (HIV1/2)	HeLa cells, human T cell line A3.01	MG132, lactacystin	Reduced	Gag processing and virus particle release	(78)
	Human CD4^+^ T cells, human CD4^+^ cell line OM-10.1	Bortezomib, lactacystin, MG132	Reduced	Infectivity of the virion and viral latency	(79)
Influenza A virus	Canine kidney cells MDCK	MG132, bortezomib	Reduced	Post fusion	(80)
Minute virus of mice^1^	Murine B cells A9	MG132, lactacystin, epoxomicin	Reduced	Post endosomal escape	(81)
Polio virus	HeLa cells	MG132, bortezomib	Reduced	Post entry (no effect on translation)	(82)
Vaccinia virus	HeLa cells	MG132, epoxomicin	Reduced	Post entry (viral genome replication; intermediate and late gene expression)	(83)
	HeLa cells	MG132, bortezomib	Reduced	Genome uncoating, replication, late viral gene expression, virus assembly	(84)

*The table summarizes viruses with known cardiac tropism and the impact of different proteasome inhibitors (bortezomib, MG132, lactacystin, MLN353, MG115, as well as, the immunoproteasome-selective inhibitor ONX 0914 (59)), of the proteasome activator PA28 (85), as well as, of the i-proteasome (cell culture and mouse studies using LMP7^−/−^ mice or cell lines obtained from these mice (47) on viral replication. CHO, chinese hamster ovary; MDCK, madin-darby canine kidney; Gag, group-specific antigen; n.r., not reported; MLN353, Millennium353 (proteasome inhibitor); ONX 0914, immunoproteasome-specific inhibitor; PA28α/β, proteasome activator α/β of 28 kDa*.

1 *Murine pathogens*.

Approximately 20 viruses have been implicated in human myocarditis and some of them interfere directly with the UPS. Among them, parvovirus B19 is detected often in endomyocardial biopsies obtained from patients with clinically suspected myocarditis (9). Parvoviruses follow multiple strategies for nuclear transport, some of them requiring active proteasomes. Replication of minute virus of mice—a murine parvovirus—is disrupted in the presence of proteasome inhibitors (81). In addition to parvoviruses, members of the herpesviridae family like human herpesvirus 6 (HHV6) are commonly detected pathogens in cardiac biopsies (9). HHV6 causes accumulation of p53 in the cytoplasm (86), and among many mechanisms regulating p53 activity, the cellular abundance of p53 is controlled by UPS-dependent turnover (87). In herpes simplex virus (HSV) infection, proteasome activity directly affects virus progeny formation. Since inhibitors of the proteasome block HSV entry at a step occurring after capsid penetration into the cytosol but prior to capsid arrival at the nuclear periphery, it was concluded that cellular proteasome activity facilitates virus entry at this early stage (74). The human cytomegalovirus (HCMV) pp71 protein stimulates quiescent cells to enter the cell cycle by targeting proteins of the retinoblastoma (Rb) family for proteasome-dependent degradation (88) and proteasome inhibitors block viral DNA replication, as well as, assembly of HCMV (76). The annual influenza virus (IV) season also calls upon some cases of IV-induced myocarditis in man. Proteasome inhibitors attenuate virus progeny formation at a post-fusion step upon influenza A virus (IAV) infection, and UPS activity is required for RNA synthesis of the virus (80). A similar function of the proteasome machinery at a post-entry step during viral replication applies to DNA replication and expression of intermediate and late genes of the vaccinia virus (83). Work is still in progress to unravel the role of the proteasome in the replication of human immunodeficiency virus (HIV). Thus far, it was shown that proteasome inhibition interferes with gag polyprotein processing, release and maturation of HIV-1 and HIV-2 (78, 79).

Although the frequency of adenovirus and coxsackie B virus detection in human myocarditis has gradually declined in adults in Western Europe during the last two decades, they are still a common cause of myocarditis in children or reported in small regional outbreaks. The adenovirus (Ad) E4 protein requires active proteasomes to promote late gene expression (69). Moreover, the Ad E1A protein regulates proteasomal activity, but is also a substrate for proteasome-mediated degradation (89). Recently, the Weinberg group established a mouse model of pediatric Ad-mediated myocarditis following intranasal infection of neonatal C57BL/6 mice with mouse adenovirus 1 (MAV-1) (90). MAV-1-myocarditis induces IFN-γ-mediated i-proteasome formation in infected heart tissue, but the catalytic activity of the β5i/LMP7 i-proteasome subunit had no effect on viral genome copy numbers in heart tissue (31). Therefore, it is unlikely that MAV-1 replication is affected by i-proteasome activity. In addition to *in vivo* models for the investigation of viral heart disease, *in vitro* studies have substantial advantages to provide information on the function of the proteasome regarding virus progeny formation. Most detailed information on the proteasome during the replicative phase of a human cardiotropic virus is available for CVB3. The McManus/Luo group was first to report a substantial suppression of CVB3 replication in HL-1 cells upon treatment with pan-specific proteasome inhibitors. This inhibitory effect was independent of the blockade of viral entry into host cells and rather attributed to reduced genome replication (70). The Luo group followed proteasome inhibition also during CVB3-induced myocarditis using A/J mice, which are known to be highly susceptible for CVB3-induced pathogenesis. In their study, MLN353 was introduced as a novel proteasome inhibitor for *in vivo* application. In contrast to the robust suppression of viral replication upon MG132 treatment in the HL-1 myxoma cell line (70), MLN353 treatment of mice did not influence virus titers (71). These somewhat controversial findings indicate that other essential pathways for CVB3 control might possibly be adversely influenced by MLN353, and this could outweigh the suppressive effect of proteasome inhibitors in cells targeted by virus infection. Our group investigated the contribution of specific proteasome subunits on the replication cycle of CVB3 *in cellulo* under one-step conditions using both HeLa cells and murine primary embryonic cardiomyocytes. PR825, as well as, ONX 0914 were applied at non-toxic concentrations to specifically block the catalytic activity of either β5 or β5i/LMP7, respectively. The CVB3 replication cycle involving the adsorption, penetration, replication of the parent virus, and release of progeny virus was not altered by the selective inhibition of these proteasome subunits (72). In addition to diverging peptidase activities of the six catalytic subunits, proteasome activity can be regulated upon binding to regulatory particles like the proteasome activator of 28 kDa (PA28). PA28-capped proteasome complexes are equipped with increased peptide hydrolysis capacity (91), and by as yet unknown mechanisms PA28 suppresses the CVB3 replication machinery (73). Altogether, a broad spectrum of various viral pathogens exploits the proteasome machinery in cells of the host organism.

## Innate immunity: how the proteasome affects the first defense wave

### Type I interferons during viral myocarditis: control by proteasome activity

During viral infection, viral RNAs and replication intermediates bind to their respective intracellular pattern recognition receptors, including Toll-like receptors (TLRs) and retinoic acid-inducible gene I (RIG-I), and, mediated by several distinct signaling pathways, this increases the production of T1IFNs [refer to (92) for a detailed review on T1IFNs in infectious disease]. T1IFNs are an effective first line of defense against viral infections and as such, a robust T1IFN response is highly beneficial to counteract early CVB3 infection in mice (93–95). Results from a pilot trial indicated a putative beneficial therapeutic influence of T1IFN substitution in patients with coxsackieviral myocarditis (96, 97). Following activation of the IFNα/β receptor (IFNAR), a diverse repertoire of antiviral proteins is expressed including protein kinase R (PKR), 2,5 oligoadenylate synthetase-like protein 2 (OASL-2), IFN-induced proteins with tetratricopeptide repeats (IFITs), as well as, IFN-stimulated genes like ISG15. The latter is an ubiquitin family protein, which is strongly induced by T1IFNs and NF-κB signaling in cardiomyocytes (98, 99), suppresses coxsackieviral replication, mitigates profoundly viral myocarditis and blocks the progression to its debilitating sequela (99).

Plasmacytoid dendritic cells (pDCs) are a major source for T1IFNs during viral myocarditis (100) and unique regarding their TLR7 or TLR9-dependent activation of IFN regulatory factor 7 (IRF7)-mediated IFNα/β production (101). Whereas, molecular accounts on the influence of ubiquitin modifications on pattern recognition receptor (PRR)-mediated signaling are available (102), less is known about the role of the different peptidase activities of the proteasome during the process from engagement of PRR to T1IFN production. Pan-specific inhibitors of the proteasome like bortezomib or carfilzomib, which target both the standard proteasome and i-proteasome, are potent suppressors of TLR9 activation in murine bone marrow cells, as well as, human peripheral blood mononuclear cells (PBMCs), but other TLR-mediated pathways like Toll/interleukin-1 receptor-domain-containing adapter-inducing IFNβ (TRIF)-mediated IRF3 activation are affected as well (63). Selective i-proteasome inhibitors assigned specifically the control of IFNα/β production in pDCs to i-proteasome peptidase activity (59, 63). Correspondingly, i-proteasome inhibition in CVB3-infected C57BL/6 (B6) mice substantially reduces T1IFN production. Thereby, i-proteasome inhibition aggravates disease parameters like viral load in B6 mice (72). On the other hand, ISGs in germline LMP7^−/−^ mouse models are as active as in wild-type controls during viral myocarditis (23). Indisputably, numerous studies indicate that the effects of T1IFN on the host response to infection are not limited to the acute, cell-intrinsic antiviral response described above. IFNα/β are also involved at various stages in the activation of adaptive immune cell responses e.g., by evolving antigen presenting DCs into a mature state (92). Similar to this, in hosts exhibiting high susceptibility for development of severe acute and chronic heart pathology like A.BY/SnJ mice, a shifted and overall significantly impaired T1IFN response (9, 100) leads to reduced DC activation and lower cross-presentation (100, 103). Genetic defects of i-proteasome subunits in mice that lead to impaired i-proteasome formation or proteasome inhibitor treatment decrease DC activation, thus, influencing the immune-stimulatory capacity of DCs as reflected by altered co-stimulatory molecule and C-C chemokine receptor 7 (CCR7) expression, as well as, cytokine production, respectively (104, 105). Thereby, i-proteasome-mediated proteolysis might directly control the antigen presentation capacity of DCs.

In contrast to the classical antiviral function of T1IFNs, there is increasing appreciation that IFNα/β can also be harmful, e.g., by triggering excessive inflammation and tissue damage (106). Likewise, IFNα/β is a classical disease-trigger of autoimmunity and auto-inflammation, and a reduced IFNα/β production as achieved upon administration of i-proteasome-selective inhibitors attenuates disease manifestation in models of lupus erythematosus (63). Defects in the DNA three prime repair exonuclease 1 (Trex1), which result in high cyclic guanosine monophosphate–adenosine monophosphate synthase (cGAS) induced IFNα/β production, lead to spontaneous inflammatory myocarditis in mice and Aicardi-Goutières syndrome in man (107, 108). Similarly, mutations in different genes encoding protein subunits of the human proteasome restrain T1IFN production, and this commences to a syndrome involving chronic atypical neutrophilic dermatosis with lipodystrophy and elevated temperature (CANDLE) (109–111).

### Effect of the proteasome for humoral innate immunity

In addition to the cellular branch of innate immunity that comprises cell-associated pattern recognition receptors, its humoral branch includes molecules such as the classic short pentraxin C-reactive protein (CRP), the long pentraxin PTX3, and complement recognition molecules (112). During viral myocarditis, PTX3 is produced mainly by monocytes and macrophages (113, 114). PTX3 promotes the engulfment of cellular debris by immune cells (115), and acts as a safeguard mechanism dampening myocardial injury induced upon pattern-associated molecular pattern (PAMP)/damage-associated molecular pattern (DAMP) signaling (112). Although, the detailed molecular aspects are unresolved, the peptidase activity of the i-proteasome controls PTX3 expression in TLR4-activated macrophages during viral myocarditis (114) and pneumococcal pneumonia (116), a function of the i-proteasome which cannot be compensated by enhanced formation of standard proteasome in LMP7^−/−^ mice (114).

### The proteasome balances protein homeostasis

Myocarditis in CVB3 (Nancy)-infected LMP7^−/−^ mice on a B6 background lacking intact i-proteasomes is not only mirrored by reduced PTX3 production (114), but it also comprises high-grade inflammation and increased cell death (23). In cells with high rate of protein synthesis e.g., in response to cytokine signaling, a reduction of translational fidelity often occurs, generating defective ribosomal products (16). Cells in general and cardiomyocytes in particular that produce higher amounts of i-proteasomes are equipped with increased proteolytic activity and can efficiently degrade defective proteins (32, 33, 117). Thereby, the i-proteasome diminishes tissue damage in mouse hearts of CVB3-infected wild-type B6 mice (23). Nevertheless, this finding in B6 mice is in clear contrast to findings made in A/J mice, which exhibit high susceptibility for virus-mediated inflammation of heart tissue (118, 119) and generally present with increased viral burden in the heart. Here, i-proteasome activity constitutes severe cytokine-mediated inflammatory heart tissue injury. I-proteasome inhibition blocks chemokine and cytokine production, and consequently reduces the appearance of misfolded proteins (72). The use of selective inhibitors targeting i-proteasome activity does not necessarily reflect the findings obtained in respective germ-line gene deficient mouse models (23, 50, 72). As an example, contrary to what was reported in LMP7^−/−^ B6 mice, inhibition of i-proteasome activity by ONX 0914 in CVB3-infected wild-type B6 mice disrupts the T1IFN defense against the invading pathogen, facilitates virus-mediated tissue damage and exacerbates PAMP/DAMP-signaling in the heart. Thereby, the production of chemokines, infiltration with immune cells, as well as, cytokine release increase (72). Such discrepancies between specific inhibitors for proteasome subunits and their knockout models might be due to a compensatory formation of standard proteasomes in LMP7^−/−^ mice (50, 59), which is not observed at a similar level in ONX 0914-treated mice.

### Innate myeloid cells: proteasome activity regulates chemokine and cytokine production

Neutrophils are the first and most abundant cell population of the host's innate immune response with well-known function in the defense against bacterial and fungal pathogens. Moreover, neutrophil recruitment in virus infection can be part of a protective strategy leading to prevention of viral disease (120). The i-proteasome influences the abundance of these cells in blood and spleen, but it controls the activation status of neutrophils as well (72, 121). Nevertheless, neutrophils have no disease modifying impact on CVB3-induced myocarditis (72, 122, 123). During myocarditis, particularly monocytes/macrophages—that emigrate the bone marrow, then sequester and differentiate in the spleen—infiltrate the infected mouse heart (23, 99). Chemokines attract these cells to the injured heart, where they are indispensable for waste removal and healing (7, 124). On the other hand, many studies have highlighted the requirement of monocytes/macrophages for the manifestation of the detrimental consequences of viral myocarditis—inflammatory injury and formation of fibrotic scar (72, 125–128). Similar to monocytes, macrophages also exacerbate inflammatory injury in infected mouse hearts (127). Monocytes and macrophages secrete pro-inflammatory and pro-fibrotic cytokines (7, 126). Therefore, molecules involved in innate immune cell mobilization and differentiation or in the control of cytokine/chemokine production by these cells present putative drug targets for future investigation. Resembling their effects on neutrophils, i-proteasome inhibitors stimulate also monocyte/macrophage emigration from the bone marrow and increase the abundance particularly of Ly6C^high^ monocytes in the spleen (72, 121), where they differentiate to macrophages under inflammatory conditions (129). These findings might be indicative for a pro-inflammatory function of the i-proteasome.

**Figure 1:**
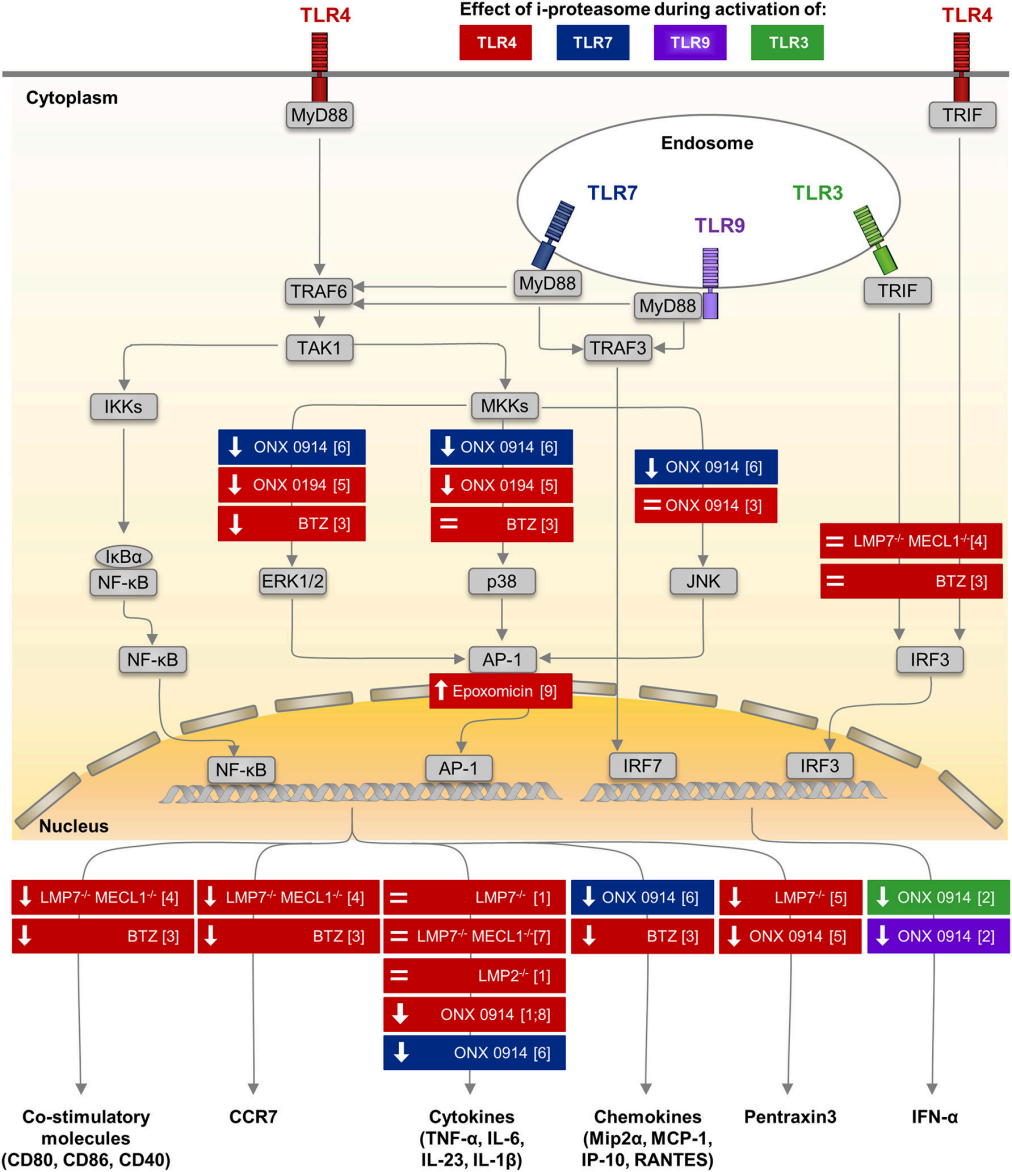
Impact of i-proteasome subunits on innate immune signaling in myeloid cells. Among many different pattern recognition receptors, TLRs are sensors of microbial antigens on monocytes/macrophages and dendritic cells. These membrane-bound receptors are located both on the cellular surface (TLR4—colored in red) and in endosomes (TLR3—green, TLR7—blue, TLR9—purple) (101). Signaling pathways down-stream of TLR4, TLR7, and TLR9 involve the common adaptor molecule MyD88 (130, 131). Upon TLR stimulation, the ubiquitin E3 ligase TRAF6 engages with the TLR/MyD88 complex and generates poly-ubiquitin scaffolds (132), thereby recruiting the TAK1 complex (133). TAK1 then activates the IKK complex, which in turn phosphorylates IκBα. Ubiquitination of IκBα marks it for degradation by the proteasome. Thereafter, NF-κB translocates into the nucleus. Simultaneously, TAK1 induces MAP kinase signaling (134), which results in the phosphorylation of ERK1/2, p38, and JNK and thereby activates the transcription factor AP-1. Both NF-κB and AP-1 induce the expression of co-stimulatory molecules (CD80, CD86, CD40) and migration signals (CCR7) on DCs, the secretion of pro-inflammatory cytokines (e.g., TNF-α, IL-6, IL-23, IL-1β), chemokines (e.g., Mip2α, MCP-1, IP-10, RANTES), and of Pentraxin3 by monocytes/macrophages (cytokines partially also by DCs). MyD88-dependent TLR7/9 signaling induces the phosphorylation of IRF7, which is a key regulator of T1IFN (IFNα, IFNβ) expression in pDCs (135). Signals from TLR3 and TLR4 are transmitted by a MyD88-independent, TRIF-dependent pathway involving activating kinases (131). Phosphorylation of IRF-3 induces translocation into the nucleus. Results obtained from *in vitro* studies, in which the impact of the different peptidase activities of the proteasome isoforms regarding to TLR signaling or the expression of effector molecules were investigated by different approaches, are summarized. Colors indicate the type of TLR stimulated to activate innate immune cells of different origin including human PBMCs, murine splenocytes, bone marrow cells and peritoneal macrophages. Each box illustrates both the model used to alter a specific peptidase activity of the proteasome—innate myeloid cells isolated from knock out mice or proteasome inhibitors with different specificity studied in innate myeloid cells, as well as, the observed effect either on the respective signaling pathway or on the production of respective effector molecules. (↓): reduced phosphorylation of a key molecule in the indicated signaling pathway or lower production of the effector molecule, = no alteration of signaling or production of the effector molecule. AP-1, activator protein 1; BTZ, bortezomib—a pan-specific proteasome inhibitor included because the i-proteasome is highly abundant in DCs (136), CCR7, C-C chemokine receptor type 7; DC, dendritic cell; ERK, extracellular signal–regulated kinases; IκBs, inhibitors of κB; IKK, IκB kinase; IP-10, interferon-gamma induced protein 10; IRF3, interferon regulatory factor 3; JNK, c-Jun N-terminal kinase; MAPK, mitogen-activated protein kinase; MCP-1, monocyte chemoattractant protein 1; Mip2α, macrophage inflammatory protein 2α; MKK, mitogen-activated protein kinase kinase; MyD88, myeloid differentiation primary response 88; NF-κB, nuclear factor-κB; ONX 0914, immunoproteasome inhibitor (59); RANTES, regulated on activation; normal T cell expressed and secreted; T1IFN, type I interferon; TAK1, transforming growth factor-β activated kinase 1; TLR, Toll-like receptor; TNF-α, tumor necrosis factor α; TRAF, TNF receptor associated factor; TRIF, TIR-domain-containing adapter inducing IFNβ. (1) (59) (2) (63) (3) (104) (4) (105) (5) (114) (6) (72) (7) (137) (8) (138) (9) (116).

In contrast, there is considerable experimental evidence from various *in vitro* and *in vivo* approaches that argues substantially against this notion and rather advocates i-proteasome-selective inhibitors as anti-inflammatory drugs e.g., for autoimmunity or to prevent transplant rejection (50, 59, 62, 65, 66). As summarized in Figure 1, selective inhibitors of the i-proteasome suppress the production of pro-inflammatory cytokines such as TNF-α and IL-6 in TLR4 and TLR7 activated immune cells. Similar results were obtained in IFNγ and TLR4 activated mouse macrophages (137), TLR4 stimulated splenocytes (59, 138), TLR4 activated PBMCs from healthy donors and patients with rheumatoid arthritis (59), as well as, TLR7 engaged macrophages (72). Consistently, in CVB3 infected A/J mice, the i-proteasome affects cytokine production also (72). Nevertheless, it needs to be recalled that under conditions where i-proteasome activity is needed for pathogen control like during *Candida albicans* or CVB3 infection of B6 mice, this influence of i-proteasome proteolysis on cytokine production seems to be outweighed by a higher PAMP burden (72, 121). In this case, the pathogen load is presumably a much stronger effector of cytokine production than the cellular content of the i-proteasome.

### Influence of proteasome peptidase activity on TLR signaling

In A/J mice, CVB3 replicates to about 10-fold increased titers in the heart in comparison to B6 mice (72). One might speculate that the overall increase in viral RNA ultimately stimulates PRR signaling in mouse hearts, thereby facilitating cytokine/chemokine production. In fact, the inflammatory response in infected heart tissue is higher in A/J mice if directly compared to B6 mice. It remains an enigma how i-proteasome catalyzed proteolysis controls PRR signaling at a molecular level. In addition, it is unclear why the i-proteasome affects differently the cardiac phenotype during MAV-1 and CVB3-induced myocarditis in B6 mice (31, 72). CVB3 as a single-stranded RNA virus is a bona fide activator of TLR7 and TLR8 (139) [in mice only TLR7 is active (140)]. Viral DNA from Ad however triggers the TLR9 pathway. Alternatively, Ad escaping the endosome reveals viral DNA complexes to the cytosolic compartment and sensors like cGAS, which acts by the stimulator of interferon genes (STING)-controlled immune pathway (141). Thereby induced signaling stimulates transcription factors like IRF7 (TLR7, TLR9) activator protein 1 (AP-1) (TLR7, TLR9), NF-κB (TLR7, TLR9, STING), and IRF3 (STING) leading to the induction of target genes that—in addition to IFNs and other ISGs - also encode pro-inflammatory cytokines and chemokines (101, 142). Therefore, we have summarized the current understanding on how the i-proteasome influences e.g., TLR mediated cellular signaling in Figure 1.

**Figure 2:**
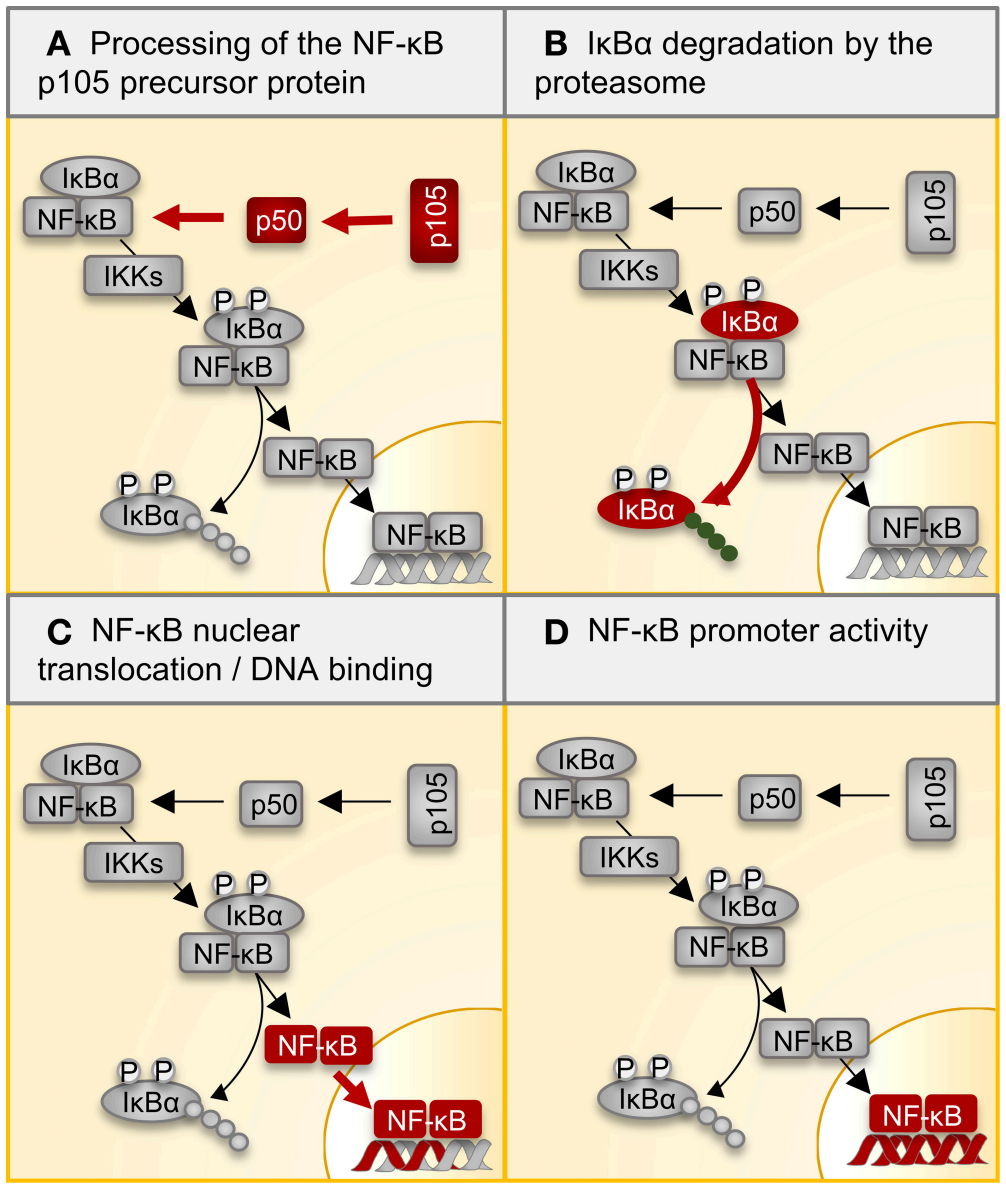
Regulation of NF-κB signaling by the proteasome. Multiple inflammatory signals result in the activation of the transcription factor NF-κB through a variety of adapter proteins and kinases. The most abundant form of the NF-κB dimer is the p50/p65 heterodimer. **(A)** The p105 precursor is processed by the proteasome, thereby liberating the NF-kB p50 subunit for dimerization with p65. IκB retains the NF-κB heterodimer in the cytoplasm. **(B)** Ligand binding to cellular receptors like TLRs activates the IKK complex, which catalyzes the phosphorylation of IκB, inducing its poly-ubiquitination and degradation by the proteasome. **(C)** Activated NF-κB translocates into the nucleus, where it **(D)** activates target gene expression. Table 2 summarizes all reported effects of i-proteasome activity on the different steps in this canonical NF-κB signaling pathway. NF-kB, nuclear factor kappa B; IκBs, inhibitors of κB; IKK, IκB kinase.

The NF-κB family of transcription factors, which acts downstream of TLR7, TLR9, and STING, plays a central role in regulation of inflammation. In the canonical pathway of NF-κB activation, the proteasome degrades IκBα, releasing the active NF-κB dimer (usually p65/p50) and allowing translocation to the nucleus (Figure 2). The impact of the different proteasome isoforms on NF-κB signaling is reported controversially (summarized in Table 2). A defective NF-κB activation as a response to reduced LMP2 expression in non-obese mice was attributed to reduced processing of the NF-κB precursor p105 (143, 145), but two different laboratories rebutted these findings (146, 150). Other data confirmed the initial findings and suggested an altered stimulation of canonical NF-κB activation by the i-proteasome in comparison to the standard proteasome. 20S i-proteasomes accelerate IκBα degradation (144), p65 nuclear translocation is lower in IFN-γ activated murine embryonic fibroblasts from LMP7^−/−^ mice (149), and LPS-activated B cells from LMP2^−/−^ degrade IκBα less efficiently than controls do (147). However, different groups revisited these aspects and novel data reported on contradictory findings arguing that the i-proteasome plays no obligatory role in the degradation of IκBα and activation of the canonical NF-κB pathway (59, 114, 137, 148). Different model systems and heterogeneous read outs for the activation of canonical NF-κB activation might attribute to these controversial findings. As illustrated in Table 2, more recent reports utilized advanced models such as primary cells obtained from different i-proteasome deficient mouse strains (LMP7^−/−^, LMP7^−/−^/Mecl-1^−/−^, LMP2^−/−^), and, more importantly, applied selective proteasome inhibitors in diverse immune and non-immune cells. Moreover, the majority of these reports focused on transcriptional activity of the canonical NF-κB pathway, whereas earlier reports indicated effects on signaling primarily at the level of p105 processing and Iκ-Bα degradation.

**Table 2:** Regulation of NF-κB signaling by the i-proteasome.

**Affected part of NF-κB pathway**	**Implicated subunit**	**Shown in/by**	**Cell type/stimulus**	**Determined by**	**References**
Processing of the NF-κB p105 precursor protein—(A)	LMP2	NOD and LMP2^−/−^ mice	Splenocytes	WB, IVP	(143)
	LMP2, MECL-1	IBD patients	Isolated proteasomes from colonic mucosa	IVP	(144)
	LMP2, LMP7	Cells lacking LMP2 and LMP7	T2 cells (human)	WB, IVP	(145)
	LMP2, LMP7	Cells lacking LMP2 and LMP7	T2 cells	WB	(146)
IκBα degradation by the proteasome—(B)	LMP2	LMP2^−/−^ mice	B cells + LPS	WB	(147)
	LMP2	NOD and LMP2^−/−^ mice	Splenocytes + TNF-α	WB	(143)
	LMP2, MECL-1	IBD patients	Isolated proteasomes from colonic mucosa	WB	(144)
	LMP2, LMP7	Cells lacking LMP2 and LMP7	T2 cells + TNF-α	WB	(145)
	LMP7	ONX 0914	Cardiomyocytes (murine) + IFN-γ/TNF-α	WB	(114)
	LMP2, LMP7	UK-101, LSK01	Lung cells H23 (human) + TNF-α	WB	(148)
	LMP7	LMP7^−/−^ mice, ONX 0914	BM macrophages + LPS	WB	(114)
	LMP2, LMP7, MECL-1	LMP7^−/−^ MECL-1^−/−^ and LMP2^−/−^ mice	Perit. Macrophages + IFN-γ/TNF-α or LPS, MEFs +IFN-γ/LPS	WB	(137)
NF-κB nuclear translocation and DNA binding—(C)	LMP2	NOD and LMP2^−/−^ mice	Splenocytes + TNF-α	EMSA	(143)
	LMP2, LMP7	Cells lacking LMP2 and LMP7	T2 cells + TNF-α	EMSA	(145)
	LMP7	LMP7^−/−^ mice	MEFs +IFN-γ/TNF-α	IF	(149)
	LMP7	LMP7^−/−^ mice	Cardiomyocytes + IFN-γ/TNF-α	TransAM® NFκB p50	(23)
	LMP7	ONX 0914	Cardiomyocytes (murine) + IFN-γ/TNF-α	WB	(114)
	LMP7	ONX 0914	BM macrophages + LPS	TransAM® NFκB p50, WB	(114)
	LMP2, LMP7	UK-101, LSK01	Lung cells H23 (human) + TNF-α	WB, IF, EMSA	(148)
	LMP2, LMP7, MECL-1	LMP7^−/−^ MECL-1^−/−^ and LMP2^−/−^ mice	MEFs +IFN-γ/TNF-α	EMSA, TransAM® NFκB p65	(137)
NF-κB promoter activity—(D)	LMP2, LMP7	Cells lacking LMP2 and LMP7	T2 cells + TNF-α	Luciferase assay	(145)
	LMP2	UK-101	Lung cells H23 + TNF-α	Luciferase assay	(148)
	LMP7	LSK01	Lung cells H23 + TNF-α	Luciferase assay	(148)
	LMP7	ONX 0914	Lung cells A549 + IFN-γ/TNF-α	Luciferase assay	(59)
	LMP7	ONX 0914	Macrophages RAW264.7 (murine) + LPS	Luciferase assay	(114)

*Known effects of the i-proteasome are summarized for each step of the NF-κB signaling pathway. These steps involve: (A) processing of the NF-κB p105 precursor protein, (B) IκBα degradation by the proteasome, (C) NF-κB nuclear translocation and DNA binding, and (D) NF-κB promoter activity, respectively (Figure 2). Results that indicate a specific role of the i-proteasome for canonical NF-κB signaling are colored in light blue. Controversial findings arguing against the notion that the i-proteasome has a specific effect on canonical NF-κB signaling are colored in dark blue. NF-kB: nuclear factor kappa B, IκBs, inhibitors of κB; T2 cells, human lymphoblast cell line defective in LMP2 and LMP7; UK-101, LMP2-specific inhibitor; LSK-01, LMP7-specific inhibitor; ONX 0914, immunoprotesome-specific inhibitor. WB, Western blotting; IF, immuno-fluorescence; EMSA, electrophoretic mobility shift assay; IVP, p105 in vitro processing assay, perit. macrophages: peritoneal macrophages; BM macrophages, bone marrow-derived macrophages; MEFs, mouse embryonic fibroblasts; LPS, lipopolysaccharide*.

Similar to TLR4-stimulated cells, cytokine/chemokine production in TLR 7 activated cells also involves MyD88 signaling, which in addition to NF-κB activates mitogen-activated protein kinase kinases (MAPKK) resulting in phosphorylation of p38, c-Jun N-terminal kinases (JNKs), and extracellular signal-regulated kinases 1/2 (ERK1/2), culminating in activation of AP-1 (101, 151). Pan-specific proteasome inhibition influences this MAPKK pathway in lipopolysaccharide (LPS)-stimulated DCs (104). Since the pool of proteasomes in DCs is mostly comprised of the i-proteasome (136), such findings are indicative for a specific effect of the i-proteasome. And indeed, data from more recent work showed that the i-proteasome controls specifically the abundance and/or activity of certain kinases, phosphatases and/or regulatory proteins involved in the complex MAPK signaling network, resulting in increased MAPK phosphorylation upon engagement of TLR4 and TLR7 (72, 114). A comprehensive system biology-based approach might be most appropriate to dissect the involved effectors that rely on functional i-proteasome activity. If and how i-proteasome activity influences mRNA transcription of genes that are under the control of IRF3, IRF8, and IRF7 is still a matter of ongoing investigation. TLR4-activated DCs from LMP7^−/−^/Mecl-1^−/−^ mice show unaltered phosphorylation of IRF3 (105). The pan-specific inhibitor of the proteasome bortezomib interferes with IRF-3 and IRF-8 activation in response to LPS in human DCs (104), suggesting a selective effect of proteasome inhibition on the IRF-3 pathway as well.

### Natural killer cells

Natural killer (NK) cells as lymphoid effectors of the rapidly acting antiviral immune response are among the first cells to sense pro-inflammatory cytokines. More than two decades ago, the importance of NK cells for CVB3 clearance and disease progression was highlighted in mice (152, 153). More recently, this pathobiological significance could be extended by providing firm evidence for a protective role of the NK cell receptor NKG2D, which upon activation triggers effective virus clearance in myocarditis (154). Knowledge regarding the impact of proteasome activity on NK cell function is incomplete and data are mainly available from tumor models. Immune surveillance of tumor cells involves a tumor necrosis factor-related apoptosis-inducing ligand (TRAIL)-mediated cytotoxic pathway used by NK cells leading to tumor cell lysis. Proteasome inhibitors like bortezomib can sensitize tumor cells to TRAIL-mediated lysis (155). If findings in tumor models might be transferable to viral myocarditis, is unknown. There is no evidence for a specific influence of the different proteasome isoforms on NK cell abundance within the inflamed heart of mice after CVB3 infection (23). Nevertheless, our current comprehension of the role of proteasome activity on NK cell function during viral myocarditis remains incomplete.

## Influence of the proteasome on establishment of adaptive immunity

The migration of NK cells and myeloid cells to the site of injury in conjunction with a considerable increase in pro-inflammatory cytokines is followed by a second wave of infiltration with CD4^+^ and to a lesser extent B and CD8^+^ T lymphocytes as well. Similar to innate immunity, virtually all knowledge about the biological function of the adaptive immune response with regard to the manifestation of viral myocarditis is based on the mouse model of CVB3-induced myocarditis. Experiments with immune-deficient mice revealed that both humoral and cellular immune responses are required to control CVB3 infection. Accordingly, mice with severe combined immunodeficiency, which lack mature B and T cell function, develop extensive myocarditis with high mortality rates (156). In this review, we expand upon established knowledge about the function of the proteasome in adaptive immunity and attempt to illuminate the implication of the different isoforms in virus control. For further details on interactions of coxsackievirus and adaptive immune system, we refer the reader to an excellent review by (15).

### Influence of CD8^+^ T cells on viral myocarditis and role of the proteasome

In contrast to the preponderant significance of B cell and CD4^+^ T cell responses for CVB3 clearance (157, 158), the pathophysiological significance of CD8^+^ T cells for CVB3 clearance and inflammatory injury is less clear. The protective function of CD8^+^ T cells (159) involves the production of cytokines like IFNγ, yet is clearly separated from the direct cytolytic effect mediated by perforin, a classic hallmark of virus-specific CD8^+^ T cells (160). CD8^+^ T cells acting by perforin cause extensive destruction of myocardial tissue (160, 161). Evidence arguing in favor of a protective function of CD8^+^ T cells during myocarditis was obtained from CD8^+^ T cell-deficient β2-microglobulin^−/−^ mice, in which injury of cardiac tissue exacerbates due to insufficient confinement of the initial viral load in the heart muscle (160). One needs to keep in mind that constitutive knockout models for perforin and β2-microglobulin do not only mirror the function of these molecules in CD8^+^ T cells. Both perforin and β2-microglobulin affect also NK cell activation and function. Nevertheless, the finding that CD8^+^ T cells restrain CVB3 in mice indicates that the virus could induce detectable CD8^+^ T cell responses. However, the Whitton group provided data that coxsackieviruses do not elicit strong CD8^+^ T cell responses. Investigation of mice infected with a recombinant CVB3 encoding known lymphocytic choriomeningitis virus (LCMV) derived CD8^+^ T cell epitopes failed to trigger a marked expansion of CD8^+^ T effector cells (162, 163). This is mainly due to the inhibition of antigen presentation by virus-induced disruption of host protein trafficking in infected cells (164, 165). The virus almost completely blocks antigen presentation via the MHC class I pathway, thereby evading CD8^+^ T cell immunity (163). Our group followed a complementary approach employing prediction tools for proteasomal cleavage sites, MHC binding studies and *in vitro* peptide processing assays with the proteasome to identify MHC class I epitopes originating from CVB3 proteins (8, 166). Concordant with the findings by the Whitton group, expansion of respective CD8^+^ T effector cells was weak in mice (8). Similarly, adoptive transfer of CD8^+^ T cells isolated from mice with CVB3 myocarditis did not affect the manifestation of viral myocarditis in recipient mice (23).

Based on these virus-specific aspects, the role for the i-proteasome with regard to induction of CD8^+^ T cell responses needs to be revisited for viral myocarditis. Following up on robust i-proteasome formation in hearts of both MAV-1 and CVB3-infected mice (30, 31), the Weinberg lab and our workgroup investigated the role of the i-proteasome concerning virus clearance in myocarditis. The i-proteasome facilitates the release of peptides harboring hydrophobic or basic C-terminal amino acids typical for MHC class I epitopes (22, 27). By facilitating such specific peptide cleavages, the i-proteasome augments the pool of antigenic peptides (40). Nevertheless, we found uniformly that the i-proteasome can be adequately compensated by its standard proteasome counterpart during viral myocarditis (23, 31). Although the i-proteasome provides an increased capacity to liberate CVB3 epitopes for MHC class I antigen presentation (40, 166), it cannot compensate for the disruption of MHC class I presentation by the virus. If detectable at all, CD8^+^ effector T cell responses remain weak during CVB3 infection (163).

### CD4^+^ T cells and antibody responses in CVB3 myocarditis: impact of the proteasome

Infections with CVB3 trigger a rapid and effective antibody response. Neutralizing antibodies appear 4 days after CVB3 infection (167) and are essential for controlling virus dissemination and clearance in the heart (158). CD4^+^ T cells activate B cells for production of protective antibodies. In contrast to MHC class I, MHC class II epitopes are presented efficiently upon infection with CVB3 and CD4^+^ T cells mature quickly into effector and later on into memory T cells (163). The proteasome is involved in multiple cellular processes needed for antibody production. As outlined above, it controls the maturation and activation of DCs (104), but the proteasome regulates also B cell function (147). The canonical pathway for MHC class II antigen presentation is located within the endolysosomal compartment and thereby spatially separated from the proteasome. However, there is also a non-canonical cytosolic pathway of MHC class II-restricted antigen processing involving proteasome-dependent peptide processing. In addition to DCs exposed to exogenous influenza and vaccinia virus (168), cancer cells present peptides on MHC class II by such non-classical antigen-processing pathways (169). It is unknown whether the cleavage site preference of the different proteasome isoforms determine a specific CD4^+^ T cell repertoire as reported for CD8^+^ T cells. To dissect the function of the i-proteasome in CVB3 myocarditis, our group applied the i-proteasome-specific inhibitor ONX 0914, and alternatively utilized LMP7^−/−^ mice. We found a strong induction of CVB3-directed immunoglobulins and neutralizing antibodies in mice lacking intact i-proteasome function (23). In fact, neutralizing antibody titers were higher in mice with ONX 0914 treatment, an observation that might be attributed to maintained survival of CD4^+^ T cells during infection in response to i-proteasome inhibition (72). The latter finding during CVB3 infection was specific for A/J mice and did not occur in B6 mice. In B6 mice, i-proteasome inhibition resulted in a reduction of lymphocyte abundance in blood and spleen at the acute phase of the disease. In fact, other groups demonstrated also a pro-survival function of the i-proteasome in T cells during viral infection with IV and LCMV (147, 170).

The fact that re-infection of B6 mice with CVB3 4 weeks after primary virus inoculation completely revokes disease manifestation emphasizes the importance of memory immune status, as well as, antibody formation during CVB3 infection (72). Upon encountering CVB3, memory T and B cells initiate cell division much more rapidly than their naive counterparts do. These data suggest that the level of MHC/peptide complex upon initial infection is sufficient to trigger memory T cells (163). In CVB3-infected B6 mice, displaying impaired i-proteasome function, adequate immune memory develops unhindered as well (23, 72). Similarly, protective immunity to MAV-1 is preserved in LMP7^−/−^ mice (31). Conclusively, the specific peptidase activities of the i-proteasome are not essential for establishment of an adaptive immune response in mouse models of viral myocarditis.

CVB-specific CD4^+^ T cells show an effector phenotype with a Th1 cytokine profile (163). In addition, A/J mice induce an autoreactive CD4^+^ T cell repertoire that contains IL-17-producing cells (11). The availability of i-proteasome selective inhibitors shed new light onto the role of the i-proteasome during CD4^+^ T cell differentiation. Under Th17 skewing conditions, inhibition of the LMP7 subunit downregulates RORγt activity leading to reduced Th17 counts, whereby lower STAT1 phosphorylation reduces IFN-γ production under Th1 skewing conditions indicative for lower Th1 counts (171). Whether or not these *in vitro* findings are relevant during viral myocarditis needs further investigation—a challenging task given the relatively weak IL-17 signal obtained from CD4^+^ T cells during acute myocarditis (11).

## Future perspectives

Several mechanisms have been proposed for CVB3-mediated myocarditis in mice, including direct virus-mediated cell damage and destruction of heart tissue in response to the action of immune effector cells (7). Being the major cellular mechanism for protein degradation, the proteasomal system adapts to augmented protein turnover by increased formation of i-proteasomes (32, 33). Based on structural information (17, 18, 29), site-specific inhibitors targeting particular subunits of the major proteasome isoforms have become available [reviewed in (51)] and our understanding about the pathophysiological role of the proteasome during CVB3-mediated myocarditis has thereby improved. In our concluding remarks, we discuss whether subunit-selective inhibitors might be applicable to suppress manifestation or progression of virus-induced cardiac injury.

Inactivation of the highly abundant β5 standard proteasome subunit in murine cardiomyocytes augments apoptosis in myocardial ischemia/reperfusion injury (172) or due to doxorubicin treatment. In contrast, even under conditions with cytokine-induced i-proteasome expression, selective i-proteasome inhibitors are advantageous in reducing cardiomyocyte death in comparison to compounds targeting either the standard or both the standard and the i-proteasome with similar efficacy (25). During viral myocarditis, i-proteasome formation and to a minor extent induction of PA28β also enhance cellular protein turnover reducing the accumulation of oxidant-damaged proteins (23, 73). The notion of a minor influence of the i-proteasome regarding the control of pathogens was supported by elimination of virus despite a reduction of T1IFN (63, 72) upon i-proteasome inhibitor treatment and induction of immune memory in CVB3 heart disease (72). This is consistent with findings for other pathogens as well (48, 59) and in addition, i-proteasome inhibitors are well tolerated in other viral infection models (31, 173). In none of these models, i-proteasome inhibition alters significantly the abundance of toxic aggregates. Most strikingly, in mice susceptible for CVB3 myocarditis, i-proteasome inhibition is highly beneficial. ONX 0914 treatment improves cardiac function and mortality by efficient suppression of cardiac and systemic chemokine and cytokine production (72).

In addition to myocarditis, experimental infection of susceptible mice with CVB3 results in severe systemic disease as well, with the pancreas being the primary and most affected organ (174). Early upon infection, mice become hypoglycemic, most likely due to pancreatitis and digestive dysfunction (175). With the release of cytokines, such systemic pathology alters the vascular tone and impairs diastolic filling as well. Systemic disease in A/J mice is reminiscent of a distributive shock in sepsis (118). Importantly, given the high abundance of i-proteasome in immune cells, i-proteasome specific inhibitors affect systemic pathology as well and this has immediate impact on the cardiac output and immune-mediated damage of heart tissue (72). Other than in the experimental mouse model, myocarditis in man usually follows a benign respiratory, gastrointestinal or urogenital infection, and pancreatitis is reported only occasionally (3). Therefore, our current understanding of i-proteasome biology during myocarditis needs further clarification. Additional research ought to elucidate the contribution of the i-proteasome once virus-mediated injury of the heart muscle has developed. In addition, we need detailed knowledge on molecular and cellular aspects of i-proteasome biology and the underlying mechanisms that contribute to the protective outcome if the i-proteasome is blocked prior to the occurrence of viral heart disease. As the i-proteasome has wide-ranging functions, toxicity and immune-related adverse effects may represent significant hurdles regarding the application of i-proteasome inhibitors. A detailed comprehension of i-proteasome function at an advanced stage of myocarditis is particularly important, because the resolution of acute CVB3 myocarditis is followed by the onset of chronic inflammation, which has been attributed to autoimmunity, as shown in genetically susceptible mice (176). Whether the i-proteasome affects also manifestation of autoimmune heart disease is unknown. Nonetheless, our current understanding of i-proteasome biology encourages a continued look at this context to define novel treatment options for viral heart disease.

## Author contributions

All authors listed have made a substantial, direct and intellectual contribution to the work, and approved it for publication.

### Conflict of interest statement

The authors declare that the research was conducted in the absence of any commercial or financial relationships that could be construed as a potential conflict of interest.

## References

[1] Cooper LT Jr. Myocarditis. N Engl J Med. (2009) 360:1526–38. 10.1056/NEJMra0800028 19357408 PMC5814110

[2] Epelman S Liu PP Mann DL. Role of innate and adaptive immune mechanisms in cardiac injury and repair. Nat Rev Immunol. (2015) 15:117–29. 10.1038/nri3800 25614321 PMC4669103

[3] Sagar S Liu PP Cooper LT Jr. Myocarditis. Lancet (2012) 379:738–47. 10.1016/S0140-6736(11)60648-X 22185868 PMC5814111

[4] Caforio AL Pankuweit S Arbustini E Basso C Gimeno-Blanes J Felix SB. Current state of knowledge on aetiology, diagnosis, management, and therapy of myocarditis: a position statement of the European Society of Cardiology Working Group on Myocardial and Pericardial Diseases. Eur Heart J. (2013) 34:2636–48. 10.1093/eurheartj/eht210 23824828

[5] Feldman AM Mcnamara D. Myocarditis. N Engl J Med. (2000) 343:1388–98. 10.1056/NEJM200011093431908 11070105

[6] Klingel K Hohenadl C Canu A Albrecht M Seemann M Mall G. Ongoing enterovirus-induced myocarditis is associated with persistent heart-muscle infection - quantitative-analysis of virus-replication, tissue-damage, and inflammation. Proc Natl Acad Sci USA. (1992) 89:314–8. 10.1073/pnas.89.1.314 1309611 PMC48227

[7] Corsten MF Schroen B Heymans S Inflammation in viral myocarditis: friend or foe? Trends Mol Med. (2012) 18:426–37. 10.1016/j.molmed.2012.05.005 22726657

[8] Jakel S Kuckelkorn U Szalay G Plotz M Textoris-Taube K Opitz E. Differential interferon responses enhance viral epitope generation by myocardial immunoproteasomes in murine enterovirus myocarditis. Am J Pathol. (2009) 175:510–8. 10.2353/ajpath.2009.090033 19590042 PMC2716952

[9] Kindermann I Kindermann M Kandolf R Klingel K Bultmann B Muller T. Predictors of outcome in patients with suspected myocarditis. Circulation (2008) 118:639–48. 10.1161/CIRCULATIONAHA.108.769489 18645053

[10] Flynn CT Kimura T Frimpong-Boateng K Harkins S Whitton JL. Immunological and pathological consequences of coxsackievirus RNA persistence in the heart. Virology (2017) 512:104–12. 10.1016/j.virol.2017.09.017 28950225 PMC5653433

[11] Gangaplara A Massilamany C Brown DM Delhon G Pattnaik AK Chapman N. Coxsackievirus B3 infection leads to the generation of cardiac myosin heavy chain-alpha-reactive CD4 T cells in A/J mice. Clin Immunol. (2012) 144:237–49. 10.1016/j.clim.2012.07.003 22854287

[12] Neumann DA Rose NR Ansari AA Herskowitz A. Induction of multiple heart autoantibodies in mice with coxsackievirus B3- and cardiac myosin-induced autoimmune myocarditis. J Immunol. (1994) 152:343–50. 8254202

[13] Rose NR. Myocarditis: infection versus autoimmunity. J Clin Immunol. (2009) 29:730–7. 10.1007/s10875-009-9339-z 19826933

[14] Kaya Z Leib C Katus HA. Autoantibodies in heart failure and cardiac dysfunction. Circ Res. (2012) 110:145–58. 10.1161/CIRCRESAHA.111.243360 22223211

[15] Kemball CC Alirezaei M Whitton JL. Type B coxsackieviruses and their interactions with the innate and adaptive immune systems. Fut Microbiol. (2010) 5:1329–47. 10.2217/fmb.10.101 20860480 PMC3045535

[16] Goldberg AL Protein degradation and protection against misfolded or damaged proteins. Nature (2003) 426:895–9. 10.1038/nature02263 14685250

[17] Groll M Ditzel L Lowe J Stock D Bochtler M Bartunik HD. Structure of 20S proteasome from yeast at 2.4 angstrom resolution. Nature (1997) 386:463–71. 10.1038/386463a0 9087403

[18] Groll M Bajorek M Kohler A Moroder L Rubin DM Huber R. A gated channel into the proteasome core particle. Nat Struct Biol. (2000) 7:1062–7. 10.1038/80992 11062564

[19] Pickart CM Cohen RE. Proteasomes and their kin: proteases in the machine age. Nat Rev Mol Cell Biol. (2004) 5:177–87. 10.1038/nrm1336 14990998

[20] Dantuma NP Lindsten K. Stressing the ubiquitin-proteasome system. Cardiovasc Res. (2010) 85:263–71. 10.1093/cvr/cvp255 19633314

[21] Dikic I. Proteasomal and autophagic degradation systems. Annu Rev Biochem. (2017) 86:193–224. 10.1146/annurev-biochem-061516-044908 28460188

[22] Gaczynska M Rock KL Goldberg AL. Gamma-interferon and expression of Mhc genes regulate peptide hydrolysis by proteasomes. Nature (1993) 365:264–7. 10.1038/365264a0 8396732

[23] Opitz E Koch A Klingel K Schmidt F Prokop S Rahnefeld A. Impairment of immunoproteasome function by beta5i/LMP7 subunit deficiency results in severe enterovirus myocarditis. PloS Pathog. (2011) 7:1–13. 10.1371/journal.ppat.1002233 21909276 PMC3164653

[24] Hallermalm K Seki K Wei C Castelli C Rivoltini L Kiessling R. Tumor necrosis factor-alpha induces coordinated changes in major histocompatibility class I presentation pathway, resulting in increased stability of class I complexes at the cell surface. Blood (2001) 98:1108–15. 10.1182/blood.V98.4.1108 11493458

[25] Spur EM Althof N Respondek D Klingel K Heuser A Overkleeft HS Inhibition of chymotryptic-like standard proteasome activity exacerbates doxorubicin-induced cytotoxicity in primary cardiomyocytes. Toxicology (2016) 353–354:34–47. 10.1016/j.tox.2016.04.010 27155237

[26] Pickering AM Koop AL Teoh CY Ermak G Grune T Davies KJA The immunoproteasome, the 20S proteasome and the PA28 alpha beta proteasome regulator are oxidative-stress-adaptive proteolytic complexes. Biochem J. (2010) 432:585–94. 10.1042/BJ20100878 20919990 PMC3133595

[27] Aki M Shimbara N Takashina M Akiyama K Kagawa S Tamura T. Interferon-gamma induces different subunit organizations and functional diversity of proteasomes. J Biochem. (1994) 115:257–69. 10.1093/oxfordjournals.jbchem.a124327 8206875

[28] Strehl B Joeris T Rieger M Visekruna A Textoris-Taube K Kaufmann SH Immunoproteasomes are essential for clearance of *Listeria monocytogenes* in nonlymphoid tissues but not for induction of bacteria-specific CD8(+) T cells. J Immunol. (2006) 177:6238–44. 10.4049/jimmunol.177.9.6238 17056553

[29] Huber EM Basler M Schwab R Heinemeyer W Kirk CJ Groettrup M. Immuno- and constitutive proteasome crystal structures reveal differences in substrate and inhibitor specificity. Cell (2012) 148:727–38. 10.1016/j.cell.2011.12.030 22341445

[30] Szalay G Meiners S Voigt A Lauber J Spieth C Speer N. Ongoing coxsackievirus myocarditis is associated with increased formation and activity of myocardial immunoproteasomes. Am J Pathol. (2006a) 168:1542–52. 10.2353/ajpath.2006.050865 16651621 PMC1606581

[31] Mccarthy MK Malitz DH Molloy CT Procario MC Greiner KE Zhang L. Interferon-dependent immunoproteasome activity during mouse adenovirus type 1 infection. Virology (2016) 498:57–68. 10.1016/j.virol.2016.08.009 27560373 PMC5045817

[32] Seifert U Bialy LP Ebstein F Bech-Otschir D Voigt A Schroter F. Immunoproteasomes preserve protein homeostasis upon interferon-induced oxidative stress. Cell (2010) 142:613–24. 10.1016/j.cell.2010.07.036 20723761

[33] Ebstein F Voigt A Lange N Warnatsch A Schroter F Prozorovski T. Immunoproteasomes are important for proteostasis in immune responses. Cell (2013) 152:935–7. 10.1016/j.cell.2013.02.018 23452842

[34] Murata S Yashiroda H Tanaka K. Molecular mechanisms of proteasome assembly. Nat Rev Mol Cell Biol. (2009) 10:104–15. 10.1038/nrm2630 19165213

[35] Murata S Sasaki K Kishimoto T Niwa S Hayashi H Takahama Y. Regulation of CD8+ T cell development by thymus-specific proteasomes. Science (2007) 316:1349–53. 10.1126/science.1141915 17540904

[36] Guillaume B Chapiro J Stroobant V Colau D Van Holle B Parvizi G. Two abundant proteasome subtypes that uniquely process some antigens presented by HLA class I molecules. Proc Natl Acad Sci USA. (2010) 107:18599–604. 10.1073/pnas.1009778107 20937868 PMC2972972

[37] Kloetzel PM Ossendorp F. Proteasome and peptidase function in MHC-class-I-mediated antigen presentation. Curr Opin Immunol. (2004) 16:76–81. 10.1016/j.coi.2003.11.004 14734113

[38] Gaczynska M Rock KL Spies T Goldberg AL. Peptidase activities of proteasomes are differentially regulated by the major histocompatibility complex-encoded genes for LMP2 and LMP7. Proc Natl Acad Sci USA. (1994) 91:9213–7. 10.1073/pnas.91.20.9213 7937744 PMC44782

[39] Sijts EJ Kloetzel PM. The role of the proteasome in the generation of MHC class I ligands and immune responses. Cell Mol Life Sci. (2011) 68:1491–502. 10.1007/s00018-011-0657-y 21387144 PMC3071949

[40] Mishto M Liepe J Textoris-Taube K Keller C Henklein P Weberruss M. Proteasome isoforms exhibit only quantitative differences in cleavage and epitope generation. Eur J Immunol. (2014) 44:3508–21. 10.1002/eji.201444902 25231383

[41] Schwarz K Van Den Broek M Kostka S Kraft R Soza A Schmidtke G Overexpression of the proteasome subunits LMP2, LMP7, and MECL-1, but not PA28 alpha/beta, enhances the presentation of an immunodominant lymphocytic choriomeningitis virus T cell epitope. J Immunol. (2000) 165:768–78. 10.4049/jimmunol.165.2.768 10878350

[42] Chen WS Norbury CC Cho YJ Yewdell JW Bennink JR. Immunoproteasomes shape immunodominance hierarchies of antiviral CD8(+) T cells at the levels of T cell repertoire and presentation of viral antigens. J Exp Med. (2001) 193:1319–26. 10.1084/jem.193.11.1319 11390439 PMC2193381

[43] Deol P Zaiss DM Monaco JJ Sijts AJ. Rates of processing determine the immunogenicity of immunoproteasome-generated epitopes. J Immunol. (2007) 178:7557–62. 10.4049/jimmunol.178.12.7557 17548590

[44] Tu L Moriya C Imai T Ishida H Tetsutani K Duan XF. Critical role for the immunoproteasome subunit LMP7 in the resistance of mice to *Toxoplasma gondii* infection. Eur J Immunol. (2009) 39:3385–94. 10.1002/eji.200839117 19830724

[45] Kincaid EZ Che JW York I Escobar H Reyes-Vargas E Delgado JC. Mice completely lacking immunoproteasomes show major changes in antigen presentation. Nat Immunol. (2012) 13:129–35. 10.1038/ni.2203 22197977 PMC3262888

[46] Ersching J Vasconcelos JR Ferreira CP Caetano BC Machado AV Bruna-Romero O. The combined deficiency of immunoproteasome subunits affects both the magnitude and quality of pathogen- and genetic vaccination-induced CD8+ T cell responses to the human protozoan parasite *Trypanosoma cruzi*. PLoS Pathog. (2016) 12:e1005593. 10.1371/journal.ppat.1005593 27128676 PMC4851296

[47] Fehling HJ Swat W Laplace C Kuhn R Rajewsky K Muller U. Mhc class-I expression in mice lacking the proteasome subunit Lmp-7. Science (1994) 265:1234–7. 10.1126/science.8066463 8066463

[48] Nussbaum AK Rodriguez-Carreno MP Benning N Botten J Whitton JL. Immunoproteasome-deficient mice mount largely normal CD8(+) T cell responses to lymphocytic choriomeningitis virus infection and DNA vaccination. J Immunol. (2005) 175:1153–60. 10.4049/jimmunol.175.2.1153 16002717

[49] Vankaer L Ashtonrickardt PG Eichelberger M Gaczynska M Nagashima K Rock KL Altered peptidase and viral-specific T-cell response in Lmp2 mutant mice. Immunity (1994) 1:533–41. 10.1016/1074-7613(94)90043-4 7600282

[50] Basler M Mundt S Muchamuel T Moll C Jiang J Groettrup M. Inhibition of the immunoproteasome ameliorates experimental autoimmune encephalomyelitis. EMBO Mol Med. (2014) 6:226–38. 10.1002/emmm.201303543 24399752 PMC3927957

[51] Kisselev AF Groettrup M. Subunit specific inhibitors of proteasomes and their potential for immunomodulation. Curr Opin Chem Biol. (2014) 23:16–22. 10.1016/j.cbpa.2014.08.012 25217863 PMC4564373

[52] Richardson PG Barlogie B Berenson J Singhal S Jagannath S Irwin D. A phase 2 study of bortezomib in relapsed, refractory myeloma. N Engl J Med. (2003) 348:2609–17. 10.1056/NEJMoa030288 12826635

[53] Richardson PG Sonneveld P Schuster MW Irwin D Stadtmauer EA Facon T Bortezomib or high-dose dexamethasone for relapsed multiple myeloma. N Eng J Med. (2005) 352:2487–98. 10.1056/NEJMoa043445 15958804

[54] Parlati F Lee SJ Aujay M Suzuki E Levitsky K Lorens JB. Carfilzomib can induce tumor cell death through selective inhibition of the chymotrypsin-like activity of the proteasome. Blood (2009) 114:3439–47. 10.1182/blood-2009-05-223677 19671918

[55] Stewart AK Rajkumar SV Dimopoulos MA Masszi T Spicka I Oriol A. Carfilzomib, lenalidomide, and dexamethasone for relapsed multiple myeloma. N Engl J Med. (2015) 372:142–52. 10.1056/NEJMoa1411321 25482145

[56] Dimopoulos MA Goldschmidt H Niesvizky R Joshua D Chng WJ Oriol A. Carfilzomib or bortezomib in relapsed or refractory multiple myeloma (ENDEAVOR): an interim overall survival analysis of an open-label, randomised, phase 3 trial. Lancet Oncol. (2017) 18:1327–37. 10.1016/S1470-2045(17)30578-8 28843768

[57] Gomes AV Zong C Edmondson RD Li X Stefani E Zhang J. Mapping the murine cardiac 26S proteasome complexes. Circul Res. (2006) 99:362–71. 10.1161/01.RES.0000237386.98506.f7 16857966

[58] De Bruin G Xin BT Kraus M Van Der Stelt M Van Der Marel GA Kisselev AF. A set of activity-based probes to visualize human (Immuno)proteasome activities. Angew Chem Int Ed Engl. (2015) 55:4199–203. 10.1002/anie.201509092 26511210

[59] Muchamuel T Basler M Aujay MA Suzuki E Kalim KW Lauer C. A selective inhibitor of the immunoproteasome subunit LMP7 blocks cytokine production and attenuates progression of experimental arthritis. Nat Med. (2009) 15:781–7. 10.1038/nm.1978 19525961

[60] Koerner J Brunner T Groettrup M. Inhibition and deficiency of the immunoproteasome subunit LMP7 suppress the development and progression of colorectal carcinoma in mice. Oncotarget (2017) 8:50873–88. 10.18632/oncotarget.15141 28881611 PMC5584212

[61] Vachharajani N Joeris T Luu M Hartmann S Pautz S Jenike E. Prevention of colitis-associated cancer by selective targeting of immunoproteasome subunit LMP7. Oncotarget (2017) 8:50447–59. 10.18632/oncotarget.14579 28881574 PMC5584149

[62] Basler M Dajee M Moll C Groettrup M Kirk CJ. Prevention of experimental colitis by a selective inhibitor of the immunoproteasome. J Immunol. (2010) 185:634–41. 10.4049/jimmunol.0903182 20525886

[63] Ichikawa HT Conley T Muchamuel T Jiang J Lee S Owen T. Beneficial effect of novel proteasome inhibitors in murine lupus via dual inhibition of type I interferon and autoantibody-secreting cells. Arthritis Rheum. (2012) 64:493–503. 10.1002/art.33333 21905015 PMC4584406

[64] Basler M Lindstrom MM Lastant JJ Bradshaw JM Owens TD Schmidt C Co-inhibition of immunoproteasome subunits LMP2 and LMP7 is required to block autoimmunity. EMBO Rep. (2018) 19:e46512 10.15252/embr.201846512 PMC6280796 30279279

[65] Sula Karreci E Fan H Uehara M Mihali AB Singh PK Kurdi AT. Brief treatment with a highly selective immunoproteasome inhibitor promotes long-term cardiac allograft acceptance in mice. Proc Natl Acad Sci USA. (2016) 113:E8425–32. 10.1073/pnas.1618548114 27956634 PMC5206568

[66] Li J Basler M Alvarez G Brunner T Kirk CJ Groettrup M. Immunoproteasome inhibition prevents chronic antibody-mediated allograft rejection in renal transplantation. Kidney Int. (2018) 93:670–80. 10.1016/j.kint.2017.09.023 29229189

[67] Banks L Pim D Thomas M. Viruses and the 26S proteasome: hacking into destruction. Trends Biochem Sci. (2003) 28:452–9. 10.1016/S0968-0004(03)00141-5 12932734

[68] Luo H. Interplay between the virus and the ubiquitin-proteasome system: molecular mechanism of viral pathogenesis. Curr Opin Virol. (2016) 17:1–10. 10.1016/j.coviro.2015.09.005 26426962 PMC7102833

[69] Corbin-Lickfett KA Bridge E. Adenovirus E4-34kDa requires active proteasomes to promote late gene expression. Virology (2003) 315:234–44. 10.1016/S0042-6822(03)00527-0 14592775

[70] Luo HL Zhang JC Cheung C Suarez A Mcmanus BM Yang DC. Proteasome inhibition reduces coxsackievirus B3 replication in murine cardiomyocytes. Am J Pathol. (2003) 163:381–5. 10.1016/S0002-9440(10)63667-X 12875959 PMC1868224

[71] Gao G Zhang JC Si XN Wong J Cheung C Mcmanus B. Proteasome inhibition attenuates coxsackievirus-induced myocardial damage in mice. Am J Physiol Heart Circul Physiol. (2008) 295:H401–8. 10.1152/ajpheart.00292.2008 18515649 PMC2494750

[72] Althof N Goetzke CC Kespohl M Voss K Heuser A Pinkert S. The immunoproteasome-specific inhibitor ONX 0914 reverses susceptibility to acute viral myocarditis. EMBO Mol Med. (2018) 10:200–18. 10.15252/emmm.201708089 29295868 PMC5801517

[73] Respondek D Voss M Kuhlewindt I Klingel K Kruger E Beling A. PA28 modulates antigen processing and viral replication during coxsackievirus B3 infection. PLoS ONE (2017) 12:e0173259. 10.1371/journal.pone.0173259 28278207 PMC5344377

[74] Delboy MG Roller DG Nicola AV. Cellular proteasome activity facilitates herpes simplex virus entry at a postpenetration step. J Virol. (2008) 82:3381–90. 10.1128/JVI.02296-07 18234803 PMC2268500

[75] La Frazia S Amici C Santoro MG. Antiviral activity of proteasome inhibitors in herpes simplex virus-1 infection: role of nuclear factor-kappaB. Antivir Ther. (2006) 11:995–1004. 17302369

[76] Kaspari M Tavalai N Stamminger T Zimmermann A Schilf R Bogner E. Proteasome inhibitor MG132 blocks viral DNA replication and assembly of human cytomegalovirus. FEBS Lett. (2008) 582:666–72. 10.1016/j.febslet.2008.01.040 18242185

[77] Prösch S Priemer C Hoflich C Liebenthaf C Babel N Kruger DH. Proteasome inhibitors: a novel tool to suppress human cytomegalovirus replication and virus-induced immune modulation. Antivir Ther. (2003) 8:555–67. 14760889

[78] Schubert U Ott DE Chertova EN Welker R Tessmer U Princiotta MF. Proteasome inhibition interferes with gag polyprotein processing, release, and maturation of HIV-1 and HIV-2. Proc Natl Acad Sci USA. (2000) 97:13057–62. 10.1073/pnas.97.24.13057 11087859 PMC27177

[79] Miller LK Kobayashi Y Chen CC Russnak TA Ron Y Dougherty JP. Proteasome inhibitors act as bifunctional antagonists of human immunodeficiency virus type 1 latency and replication. Retrovirology (2013) 10:120. 10.1186/1742-4690-10-120 24156270 PMC4015732

[80] Widjaja I De Vries E Tscherne DM Garcia-Sastre A Rottier PJ De Haan CA. Inhibition of the ubiquitin-proteasome system affects influenza A virus infection at a postfusion step. J Virol. (2010) 84:9625–31. 10.1128/JVI.01048-10 20631148 PMC2937638

[81] Ros C Burckhardt CJ Kempf C. Cytoplasmic trafficking of minute virus of mice: low-pH requirement, routing to late endosomes, and proteasome interaction. J Virol. (2002) 76:12634–45. 10.1128/JVI.76.24.12634-12645.2002 12438589 PMC136711

[82] Neznanov N Dragunsky EM Chumakov KM Neznanova L Wek RC Gudkov AV. Different effect of proteasome inhibition on vesicular stomatitis virus and poliovirus replication. PLoS ONE (2008) 3:e1887. 10.1371/journal.pone.0001887 18382670 PMC2268745

[83] Satheshkumar PS Anton LC Sanz P Moss B. Inhibition of the ubiquitin-proteasome system prevents vaccinia virus DNA replication and expression of intermediate and late genes. J Virol. (2009) 83:2469–79. 10.1128/JVI.01986-08 19129442 PMC2648259

[84] Mercer J Snijder B Sacher R Burkard C Bleck CK Stahlberg H. RNAi screening reveals proteasome- and Cullin3-dependent stages in vaccinia virus infection. Cell Rep. (2012) 2:1036–47. 10.1016/j.celrep.2012.09.003 23084750

[85] Murata S Udono H Tanahashi N Hamada N Watanabe K Adachi K Immunoproteasome assembly and antigen presentation in mice lacking both PA28 alpha and PA28 beta. Embo J. (2001) 20:5898–907. 10.1093/emboj/20.21.5898 11689430 PMC125708

[86] Takemoto M Mori Y Ueda K Kondo K Yamanishi K. Productive human herpesvirus 6 infection causes aberrant accumulation of p53 and prevents apoptosis. J Gen Virol. (2004) 85:869–79. 10.1099/vir.0.19626-0 15039530

[87] Scheffner M Werness BA Huibregtse JM Levine AJ Howley PM. The E6 oncoprotein encoded by human papillomavirus types 16 and 18 promotes the degradation of p53. Cell (1990) 63:1129–36. 10.1016/0092-8674(90)90409-8 2175676

[88] Kalejta RF Shenk T. Proteasome-dependent, ubiquitin-independent degradation of the Rb family of tumor suppressors by the human cytomegalovirus pp71 protein. Proc Natl Acad Sci USA. (2003) 100:3263–8. 10.1073/pnas.0538058100 12626766 PMC152280

[89] Turnell AS Grand RJ Gorbea C Zhang X Wang W Mymryk JS. Regulation of the 26S proteasome by adenovirus E1A. Embo J. (2000) 19:4759–73. 10.1093/emboj/19.17.4759 10970867 PMC302057

[90] Mccarthy MK Procario MC Twisselmann N Wilkinson JE Archambeau AJ Michele DE. Proinflammatory effects of interferon gamma in mouse adenovirus 1 myocarditis. J Virol. (2015) 89:468–79. 10.1128/JVI.02077-14 25320326 PMC4301126

[91] Cascio P. PA28alphabeta: the enigmatic magic ring of the proteasome? Biomolecules (2014) 4:566–84. 10.3390/biom4020566 24970231 PMC4101498

[92] Mcnab F Mayer-Barber K Sher A Wack A O'garra A. Type I interferons in infectious disease. Nat Rev Immunol. (2015) 15:87–103. 10.1038/nri3787 25614319 PMC7162685

[93] Wessely R Klingel K Knowlton KU Kandolf R. Cardioselective infection with coxsackievirus B3 requires intact type I interferon signaling - implications for mortality and early viral replication. Circulation (2001) 103:756–61. 10.1161/01.CIR.103.5.756 11156890

[94] Deonarain R Cerullo D Fuse K Liu PP Fish EN. Protective role for interferon-beta in coxsackievirus B3 infection. Circulation (2004) 110:3540–3. 10.1161/01.CIR.0000136824.73458.20 15249500

[95] Althof N Harkins S Kemball CC Flynn CT Alirezaei M Whitton JL. *In vivo* ablation of type I interferon receptor from cardiomyocytes delays coxsackieviral clearance and accelerates myocardial disease. J Virol. (2014) 88:5087–99. 10.1128/JVI.00184-14 24574394 PMC3993796

[96] Kuhl U Pauschinger M Schwimmbeck PL Seeberg B Lober C Noutsias M. Interferon-beta treatment eliminates cardiotropic viruses and improves left ventricular function in patients with myocardial persistence of viral genomes and left ventricular dysfunction. Circulation (2003) 107:2793–8. 10.1161/01.CIR.0000072766.67150.51 12771005

[97] Kuhl U Lassner D Von SJ Poller W Schultheiss HP. Interferon-Beta improves survival in enterovirus-associated cardiomyopathy. J Am Coll Cardiol. (2012) 60:1295–6. 10.1016/j.jacc.2012.06.026 23017536

[98] Maier HJ Schips TG Wietelmann A Kruger M Brunner C Sauter M. Cardiomyocyte-specific IkappaB kinase (IKK)/NF-kappaB activation induces reversible inflammatory cardiomyopathy and heart failure. Proc Natl Acad Sci USA. (2012) 109:11794–9. 10.1073/pnas.1116584109 22753500 PMC3406816

[99] Rahnefeld A Klingel K Schuermann A Diny NL Althof N Lindner A. Ubiquitin-like protein ISG15 (interferon-stimulated gene of 15 kDa) in host defense against heart failure in a mouse model of virus-induced cardiomyopathy. Circulation (2014) 130:1589–600. 10.1161/CIRCULATIONAHA.114.009847 25165091

[100] Weinzierl AO Szalay G Wolburg H Sauter M Rarnmensee HG Kandolf R. Effective chemokine secretion by dendritic cells and expansion of cross-presenting CD4(-)/CD8(+) dendritic cells define a protective phenotype in the mouse model of coxsackievirus myocarditis. J Virol. (2008) 82:8149–60. 10.1128/JVI.00047-08 18550677 PMC2519575

[101] Blasius AL Beutler B. Intracellular toll-like receptors. Immunity (2010) 32:305–15. 10.1016/j.immuni.2010.03.012 20346772

[102] Heaton SM Borg NA Dixit VM. Ubiquitin in the activation and attenuation of innate antiviral immunity. J Exp Med. (2016) 213:1–13. 10.1084/jem.20151531 26712804 PMC4710203

[103] Rahnefeld A Ebstein F Albrecht N Opitz E Kuckelkorn U Stangl K. Antigen-presentation capacity of dendritic cells is impaired in ongoing enterovirus myocarditis. Eur J Immunol. (2011) 41:2774–81. 10.1002/eji.201041039 21630249

[104] Nencioni A Schwarzenberg K Brauer KM Schmidt SM Ballestrero A Grunebach F. Proteasome inhibitor bortezomib modulates TLR4-induced dendritic cell activation. Blood (2006) 108:551–8. 10.1182/blood-2005-08-3494 16537813

[105] De Verteuil DA Rouette A Hardy MP Lavallee S Trofimov A Gaucher E. Immunoproteasomes shape the transcriptome and regulate the function of dendritic cells. J Immunol. (2014) 193:1121–32. 10.4049/jimmunol.1400871 24958905

[106] Davidson S Crotta S Mccabe TM Wack A. Pathogenic potential of interferon alphabeta in acute influenza infection. Nat Commun. (2014) 5:3864. 10.1038/ncomms4864 24844667 PMC4033792

[107] Stetson DB Ko JS Heidmann T Medzhitov R. Trex1 prevents cell-intrinsic initiation of autoimmunity. Cell (2008) 134:587–98. 10.1016/j.cell.2008.06.032 18724932 PMC2626626

[108] Gray EE Treuting PM Woodward JJ Stetson DB. Cutting edge: cGAS is required for lethal autoimmune disease in the Trex1-deficient mouse model of aicardi-goutieres syndrome. J Immunol. (2015) 195:1939–43. 10.4049/jimmunol.1500969 26223655 PMC4546858

[109] Agarwal AK Xing C Demartino GN Mizrachi D Hernandez MD Sousa AB PSMB8 encoding the beta 5i proteasome subunit is mutated in joint contractures, muscle atrophy, microcytic anemia, and panniculitis-induced lipodystrophy syndrome. Am J Hum Genet. (2010) 87:866–72. 10.1016/j.ajhg.2010.10.031 21129723 PMC2997366

[110] Kitamura A Maekawa Y Uehara H Izumi K Kawachi I Nishizawa M. A mutation in the immunoproteasome subunit PSMB8 causes autoinflammation and lipodystrophy in humans. J Clin Invest. (2011) 121:4150–60. 10.1172/JCI58414 21881205 PMC3195477

[111] Brehm A Liu Y Sheikh A Marrero B Omoyinmi E Zhou Q. Additive loss-of-function proteasome subunit mutations in CANDLE/PRAAS patients promote type I IFN production. J Clin Invest. (2015) 125:4196–211. 10.1172/JCI81260 26524591 PMC4639987

[112] Magrini E Mantovani A Garlanda C. The dual complexity of PTX3 in health and disease: a balancing act? Trends Mol Med. (2016) 22:497–510. 10.1016/j.molmed.2016.04.007 27179743 PMC5414840

[113] Nebuloni M Pasqualini F Zerbi P Lauri E Mantovani A Vago L. PTX3 expression in the heart tissues of patients with myocardial infarction and infectious myocarditis. Cardiovasc Pathol. (2011) 20:e27–35. 10.1016/j.carpath.2010.02.005 20356766

[114] Paeschke A Possehl A Klingel K Voss M Voss K Kespohl M. The immunoproteasome controls the availability of the cardioprotective pattern recognition molecule Pentraxin3. Eur J Immunol. (2016) 46:619–33. 10.1002/eji.201545892 26578407

[115] Rovere P Peri G Fazzini F Bottazzi B Doni A Bondanza A. The long pentraxin PTX3 binds to apoptotic cells and regulates their clearance by antigen-presenting dendritic cells. Blood (2000) 96:4300–6. Available online at: www.bloodjournal.org/ 11110705

[116] Kirschner F Reppe K Andresen N Witzenrath M Ebstein F Kloetzel PM. Proteasome beta5i subunit deficiency affects opsonin synthesis and aggravates pneumococcal pneumonia. PLoS ONE (2016) 11:e0153847. 10.1371/journal.pone.0153847 27100179 PMC4839637

[117] Yun YS Kim KH Tschida B Sachs Z Noble-Orcutt KE Moriarity BS. mTORC1 coordinates protein synthesis and immunoproteasome formation via PRAS40 to prevent accumulation of protein stress. Mol Cell (2016) 61:625–39. 10.1016/j.molcel.2016.01.013 26876939 PMC4870089

[118] Chow LH Gauntt CJ Mcmanus BM. Differential effects of myocarditic variants of Coxsackievirus B3 in inbred mice. A pathologic characterization of heart tissue damage. Lab Invest. (1991) 64:55–64. 1990209

[119] Opavsky MA Martino T Rabinovitch M Penninger J Richardson C Petric M. Enhanced ERK-1/2 activation in mice susceptible to coxsackievirus-induced myocarditis. J Clin Invest. (2002) 109:1561–9. 10.1172/JCI0213971 12070303 PMC151008

[120] Jenne CN Kubes P Virus-induced NETs–critical component of host defense or pathogenic mediator? PLoS Pathog. (2015) 11:e1004546 10.1371/journal.ppat.1004546 25569217 PMC4287541

[121] Mundt S Basler M Buerger S Engler H Groettrup M. Inhibiting the immunoproteasome exacerbates the pathogenesis of systemic *Candida albicans* infection in mice. Sci Rep. (2016a) 6:19434. 10.1038/srep19434 26776888 PMC4726078

[122] Hiraoka Y Kishimoto C Takada H Suzaki N Shiraki K. Colony-stimulating factors and coxackievirus B3 myocarditis in mice: macrophage colony-stimulating factor suppresses acute myocarditis with increasing interferon-alpha. Am Heart J. (1995) 130:1259–64. 10.1016/0002-8703(95)90152-3 7484779

[123] Xu D Wang P Yang J Qian Q Li M Wei L. Gr-1+ cells other than Ly6G+ neutrophils limit virus replication and promote myocardial inflammation and fibrosis following coxsackievirus B3 infection of mice. Front Cell Infect Microbiol. (2018) 8:157. 10.3389/fcimb.2018.00157 29868513 PMC5962688

[124] Jakubzick CV Randolph GJ Henson PM. Monocyte differentiation and antigen-presenting functions. Nat Rev Immunol. (2017) 17:349–62. 10.1038/nri.2017.28 28436425

[125] Goser S Ottl R Brodner A Dengler TJ Torzewski J Egashira K. Critical role for monocyte chemoattractant protein-1 and macrophage inflammatory protein-1alpha in induction of experimental autoimmune myocarditis and effective anti-monocyte chemoattractant protein-1 gene therapy. Circulation (2005) 112:3400–7. 10.1161/CIRCULATIONAHA.105.572396 16316965

[126] Zimmermann O Homann JM Bangert A Muller AM Hristov G Goeser S. Successful use of mRNA-nucleofection for overexpression of interleukin-10 in murine monocytes/macrophages for anti-inflammatory therapy in a murine model of autoimmune myocarditis. J Am Heart Assoc. (2012) 1:e003293. 10.1161/JAHA.112.003293 23316321 PMC3540678

[127] Jaquenod De Giusti C Ure AE Rivadeneyra L Schattner M Gomez RM. Macrophages and galectin 3 play critical roles in CVB3-induced murine acute myocarditis and chronic fibrosis. J Mol Cell Cardiol. (2015) 85:58–70. 10.1016/j.yjmcc.2015.05.010 26002282

[128] Leuschner F Courties G Dutta P Mortensen LJ Gorbatov R Sena B. Silencing of CCR2 in myocarditis. Eur Heart J. (2015) 36:1478–88. 10.1093/eurheartj/ehu225 24950695 PMC4465633

[129] Ginhoux F Jung S. Monocytes and macrophages: developmental pathways and tissue homeostasis. Nat Rev Immunol. (2014) 14:392–404. 10.1038/nri3671 24854589

[130] Takeda K Akira S. TLR signaling pathways. Semin Immunol. (2004) 16:3–9. 10.1016/j.smim.2003.10.003 14751757

[131] Kawai T Akira S. The role of pattern-recognition receptors in innate immunity: update on Toll-like receptors. Nat Immunol. (2010) 11:373–84. 10.1038/ni.1863 20404851

[132] Deng L Wang C Spencer E Yang L Braun A You J. Activation of the IkappaB kinase complex by TRAF6 requires a dimeric ubiquitin-conjugating enzyme complex and a unique polyubiquitin chain. Cell (2000) 103:351–61. 10.1016/S0092-8674(00)00126-4 11057907

[133] Ajibade AA Wang HY Wang RF. Cell type-specific function of TAK1 in innate immune signaling. Trends Immunol. (2013) 34:307–16. 10.1016/j.it.2013.03.007 23664135

[134] Arthur JS Ley SC. Mitogen-activated protein kinases in innate immunity. Nat Rev Immunol. (2013) 13:679–92. 10.1038/nri3495 23954936

[135] Akira S Uematsu S Takeuchi O. Pathogen recognition and innate immunity. Cell (2006) 124:783–801. 10.1016/j.cell.2006.02.015 16497588

[136] Macagno A Kuehn L De Giuli R Groettrup M Pronounced up-regulation of the PA28 alpha/beta proteasome regulator but little increase in the steady-state content of immunoproteasome during dendritic cell maturation. Eur J Immunol. (2001) 31:3271–80. 10.1002/1521-4141(200111)31:113.0.CO;2-2 11745344

[137] Bitzer A Basler M Krappmann D Groettrup M. Immunoproteasome subunit deficiency has no influence on the canonical pathway of NF-kappaB activation. Mol Immunol. (2017) 83:147–53. 10.1016/j.molimm.2017.01.019 28157553

[138] Basler M Beck U Kirk CJ Groettrup M. The antiviral immune response in mice devoid of immunoproteasome activity. J Immunol. (2011) 187:5548–57. 10.4049/jimmunol.1101064 22013127

[139] Triantafilou K Orthopoulos G Vakakis E Ahmed MA Golenbock DT Lepper PM. Human cardiac inflammatory responses triggered by Coxsackie B viruses are mainly Toll-like receptor (TLR) 8-dependent. Cell Microbiol. (2005) 7:1117–26. 10.1111/j.1462-5822.2005.00537.x 16008579

[140] Jurk M Heil F Vollmer J Schetter C Krieg AM Wagner H. Human TLR7 or TLR8 independently confer responsiveness to the antiviral compound R-848. Nat Immunol. (2002) 3:499. 10.1038/ni0602-499 12032557

[141] Lam E Stein S Falck-Pedersen E. Adenovirus detection by the cGAS/STING/TBK1 DNA sensing cascade. J Virol. (2014) 88:974–81. 10.1128/JVI.02702-13 24198409 PMC3911663

[142] Barber GN. STING: infection, inflammation and cancer. Nat Rev Immunol. (2015) 15:760–70. 10.1038/nri3921 26603901 PMC5004891

[143] Hayashi T Faustman D NOD mice are defective in proteasome production and activation of NF-kappa B. Mol Cell Biol. (1999) 19:8646–59. 10.1128/MCB.19.12.8646 10567588 PMC85003

[144] Visekruna A Joeris T Seidel D Kroesen A Loddenkemper C Zeitz M Proteasome-mediated degradation of I kappa B alpha and processing of p105 in Crohn disease and ulcerative colitis. J Clin Invest. (2006) 116:3195–203. 10.1172/JCI28804 17124531 PMC1654202

[145] Hayashi T Faustman D Essential role of human leukocyte antigen-encoded proteasome subunits in NF-kappa B activation and prevention of tumor necrosis factor-alpha-induced apoptosis. J Biol Chem. (2000) 275:5238–47. 10.1074/jbc.275.7.5238 10671572

[146] Runnels HA Watkins WA Monaco JJ. LMP2 expression and proteasome activity in NOD mice. Nat Med. (2000) 6:1064–5. 10.1038/80349 11017112

[147] Hensley SE Zanker D Dolan BP David A Hickman HD Embry AC. Unexpected role for the immunoproteasome subunit LMP2 in antiviral humoral and innate immune responses. J Immunol. (2010) 184:4115–22. 10.4049/jimmunol.0903003 20228196 PMC2941094

[148] Jang ER Lee NR Han S Wu Y Sharma LK Carmony KC. Revisiting the role of the immunoproteasome in the activation of the canonical NF-kappaB pathway. Mol Biosyst. (2012) 8:2295–302. 10.1039/c2mb25125f 22722901 PMC3462293

[149] Schmidt N Gonzalez E Visekruna A Kuhl AA Loddenkemper C Mollenkopf H. Targeting the proteasome: partial inhibition of the proteasome by bortezomib or deletion of the immunosubunit LMP7 attenuates experimental colitis. Gut (2010) 59:896–906. 10.1136/gut.2009.203554 20581238

[150] Kessler BM Lennon-Dumenil AM Shinohara ML Lipes MA Ploegh HL. LMP2 expression and proteasome activity in NOD mice. Nat Med. (2000) 6:1064. 10.1038/80346 11017111

[151] Ronkina N Kotlyarov A Dittrich-Breiholz O Kracht M Hitti E Milarski K. The mitogen-activated protein kinase (MAPK)-activated protein kinases MK2 and MK3 cooperate in stimulation of tumor necrosis factor biosynthesis and stabilization of p38 MAPK. Mol Cell Biol. (2007) 27:170–81. 10.1128/MCB.01456-06 17030606 PMC1800641

[152] Godeny EK Gauntt CJ. Involvement of natural killer cells in coxsackievirus B3-induced murine myocarditis. J Immunol. (1986) 137:1695–702. 3018079

[153] Godeny EK Gauntt CJ. Murine natural killer cells limit coxsackievirus B3 replication. J Immunol. (1987) 139:913–8. 3036947

[154] Klingel K Fabritius C Sauter M Goldner K Stauch D Kandolf R. The activating receptor NKG2D of natural killer cells promotes resistance against enterovirus-mediated inflammatory cardiomyopathy. J Pathol. (2014) 234:164–77. 10.1002/path.4369 24797160

[155] Hallett WH Ames E Motarjemi M Barao I Shanker A Tamang DL. Sensitization of tumor cells to NK cell-mediated killing by proteasome inhibition. J Immunol. (2008) 180:163–70. 10.4049/jimmunol.180.1.163 18097016

[156] Chow LH Beisel KW Mcmanus BM. Enteroviral infection of mice with severe combined immunodeficiency. Evidence for direct viral pathogenesis of myocardial injury. Lab Invest. (1992) 66:24–31. 1309927

[157] Leipner C Borchers M Merkle I Stelzner A. Coxsackievirus B3-induced myocarditis in MHC class II-deficient mice. J Hum Virol. (1999) 2:102–14. 10225212

[158] Mena I Perry CM Harkins S Rodriguez F Gebhard J Whitton JL. The role of B lymphocytes in coxsackievirus B3 infection. Am J Pathol. (1999) 155:1205–15. 10.1016/S0002-9440(10)65223-6 10514403 PMC1867001

[159] Henke A Huber S Stelzner A Whitton JL. The Role of Cd8(+) T-Lymphocytes in Coxsackievirus B3-Induced Myocarditis. J Virol. (1995) 69:6720–8. 7474082 10.1128/jvi.69.11.6720-6728.1995 PMC189582

[160] Klingel K Schnorr JJ Sauter M Szalay G Kandolf R beta 2-microglobulin-associated regulation of interferon-gamma and virus-specific immunoglobulin G confer resistance against the development of chronic coxsackievirus myocarditis. Am J Pathol. (2003) 162:1709–20. 10.1016/S0002-9440(10)64305-2 12707055 PMC1851178

[161] Gebhard JR Perry CM Harkins S Lane T Mena I Asensio VC. Coxsackievirus B3-induced myocarditis: perforin exacerbates disease, but plays no detectable role in virus clearance. Am J Pathol. (1998) 153:417–28. 10.1016/S0002-9440(10)65585-X 9708802 PMC1852975

[162] Kemball CC Harkins S Whitton JL. Enumeration and functional evaluation of virus-specific CD4(+) and CD8(+) T cells in lymphoid and peripheral sites of coxsackievirus B3 infection. J Virol. (2008) 82:4331–42. 10.1128/JVI.02639-07 18305030 PMC2293035

[163] Kemball CC Harkins S Whitmire JK Flynn CT Feuer R Whitton JL. Coxsackievirus B3 inhibits antigen presentation *in vivo*, exerting a profound and selective effect on the MHC class I pathway. PLoS Pathog. (2009) 5:e1000618. 10.1371/journal.ppat.1000618 19834548 PMC2757675

[164] Cornell CT Kiosses WB Harkins S Whitton JL. Inhibition of protein trafficking by coxsackievirus B3: multiple viral proteins target a single organelle. J Virol. (2006) 80:6637–47. 10.1128/JVI.02572-05 16775351 PMC1488957

[165] Cornell CT Mosses WB Harkins S Whitton L. Coxsackievirus B3 proteins directionally complement each other to downregulate surface major histocompatibility complex class I. J Virol. (2007) 81:6785–97. 10.1128/JVI.00198-07 17442717 PMC1933326

[166] Voigt A Jakel S Textoris-Taube K Keller C Drung I Szalay G. Generation of *in silico* predicted coxsackievirus B3-derived MHC class I epitopes by proteasomes. Amino Acids (2010) 39:243–55. 10.1007/s00726-009-0434-5 19997756

[167] Szalay G Sauter M Hald J Weinzierl A Kandolf R Klingel K. Sustained nitric oxide synthesis contributes to immunopathology in ongoing myocarditis attributable to interleukin-10 disorders. Am J Pathol. (2006b) 169:2085–93. 10.2353/ajpath.2006.060350 17148671 PMC1762471

[168] Tewari MK Sinnathamby G Rajagopal D Eisenlohr LC. A cytosolic pathway for MHC class II-restricted antigen processing that is proteasome and TAP dependent. Nat Immunol. (2005) 6:287–94. 10.1038/ni1171 15711549

[169] Matsuzaki J Tsuji T Luescher I Old LJ Shrikant P Gnjatic S. Nonclassical antigen-processing pathways are required for MHC class II-restricted direct tumor recognition by NY-ESO-1-specific CD4(+) T cells. Cancer Immunol Res. (2014) 2:341–50. 10.1158/2326-6066.CIR-13-0138 24764581 PMC4004114

[170] Moebius J Van Den Broek M Groettrup M Basler M. Immunoproteasomes are essential for survival and expansion of T cells in virus-infected mice. Eur J Immunol. (2010) 40:3439–49. 10.1002/eji.201040620 21108466

[171] Kalim KW Basler M Kirk CJ Groettrup M. Immunoproteasome subunit LMP7 deficiency and inhibition suppresses Th1 and Th17 but enhances regulatory T cell differentiation. J Immunol. (2012) 189:4182–93. 10.4049/jimmunol.1201183 22984077

[172] Tian Z Zheng H Li J Li Y Su H Wang X. Genetically induced moderate inhibition of the proteasome in cardiomyocytes exacerbates myocardial ischemia-reperfusion injury in mice. Circ Res. (2012) 111:532–42. 10.1161/CIRCRESAHA.112.270983 22740087 PMC3426260

[173] Mundt S Engelhardt B Kirk CJ Groettrup M Basler M. Inhibition and deficiency of the immunoproteasome subunit LMP7 attenuates LCMV-induced meningitis. Eur J Immunol. (2016b) 46:104–13. 10.1002/eji.201545578 26464284

[174] Horwitz MS La Cava A Fine C Rodriguez E Ilic A Sarvetnick N. Pancreatic expression of interferon-gamma protects mice from lethal coxsackievirus B3 infection and subsequent myocarditis. Nat Med. (2000) 6:693–7. 10.1038/76277 10835688

[175] Mena I Fischer C Gebhard JR Perry CM Harkins S Whitton JL. Coxsackievirus infection of the pancreas: evaluation of receptor expression, pathogenesis, and immunopathology. Virology (2000) 271:276–88. 10.1006/viro.2000.0332 10860882

[176] Esfandiarei M Mcmanus BM. Molecular biology and pathogenesis of viral myocarditis. Annu Rev Pathol. (2008) 3:127–55. 10.1146/annurev.pathmechdis.3.121806.151534 18039131

